# Transcriptome Analysis of Sunflower Genotypes with Contrasting Oxidative Stress Tolerance Reveals Individual- and Combined- Biotic and Abiotic Stress Tolerance Mechanisms

**DOI:** 10.1371/journal.pone.0157522

**Published:** 2016-06-17

**Authors:** Vemanna S. Ramu, Anjugam Paramanantham, Venkategowda Ramegowda, Basavaiah Mohan-Raju, Makarla Udayakumar, Muthappa Senthil-Kumar

**Affiliations:** 1 National Institute of Plant Genome Research, Aruna Asaf Ali Marg, New Delhi, India; 2 Department of Crop Physiology, University of Agricultural Sciences, Bangalore, India; CSIR-National Botanical Research Institute, INDIA

## Abstract

In nature plants are often simultaneously challenged by different biotic and abiotic stresses. Although the mechanisms underlying plant responses against single stress have been studied considerably, plant tolerance mechanisms under combined stress is not understood. Also, the mechanism used to combat independently and sequentially occurring many number of biotic and abiotic stresses has also not systematically studied. From this context, in this study, we attempted to explore the shared response of sunflower plants to many independent stresses by using meta-analysis of publically available transcriptome data and transcript profiling by quantitative PCR. Further, we have also analyzed the possible role of the genes so identified in contributing to combined stress tolerance. Meta-analysis of transcriptomic data from many abiotic and biotic stresses indicated the common representation of oxidative stress responsive genes. Further, menadione-mediated oxidative stress in sunflower seedlings showed similar pattern of changes in the oxidative stress related genes. Based on this a large scale screening of 55 sunflower genotypes was performed under menadione stress and those contrasting in oxidative stress tolerance were identified. Further to confirm the role of genes identified in individual and combined stress tolerance the contrasting genotypes were individually and simultaneously challenged with few abiotic and biotic stresses. The tolerant hybrid showed reduced levels of stress damage both under combined stress and few independent stresses. Transcript profiling of the genes identified from meta-analysis in the tolerant hybrid also indicated that the selected genes were up-regulated under individual and combined stresses. Our results indicate that menadione-based screening can identify genotypes not only tolerant to multiple number of individual biotic and abiotic stresses, but also the combined stresses.

## Introduction

Sunflower (*‎Helianthus annuus*) is one of the most important oilseed crops worldwide. Sunflower growing regions are characterized by constant occurrence of not only multiple individual biotic and abiotic stresses, but also simultaneous drought, pathogen infection and temperature stresses resulting in substantial loss of crop productivity [1–4]. Adding to this, recent climate changes lead to unpredictable rainfall pattern, temperature and pathogen spread [5–8]. This lead to increased interaction of pathogens with different abiotic stresses in the plant interphase [9–10]. Research in the past had largely focused on understanding plant responses to individual stresses with a limited emphasis on combined stresses [11–15]. Importantly, the available combined stress related literature indicates both shared and unique physiological and molecular responses of plants between combined and individual stresses [9, 16–18]. Therefore, uncovering the shared mechanisms using information from large number of individual stress based studies will be useful for understanding the role of commonly regulated genes under combined and individual stresses.

Transcript profiling data from drought, salt, abscisic acid (ABA), several fungal pathogens, reactive oxygen species (ROS) and cold stress treated sunflower plants are available [19–24]. Such transcriptome profiling from individual stresses such as drought and low temperature has been used to unravel the pathways associated with multiple individual stresses [25, 26]. In spite of large scale transcriptome data available from different individual stresses, a comprehensive effort to identify commonly regulated genes has not yet been made. These shared responses might reveal complex signaling networks and pathways [27, 28] facilitating understanding of both individual and combined stress tolerance mechanisms. From this direction meta-analysis of available data is useful. Recently, meta-analysis of microarray data from rice (*Oryza sativa*) and *Arabidopsis thaliana* exposed to drought and bacterial stress identified several commonly regulated stress-responsive genes [29, 30]. In a similar study in rice and *A*. *thaliana* plants exposed to drought and bacterial pathogen, ~3100 and 900 differentially expressed genes were identified respectively. About 38.5% and 28.7% differential genes were common to drought and bacterial stresses in rice and *A*. *thaliana*, respectively [31]. A large number of commonly regulated genes belonged to ROS mediated signaling and free radical scavenging pathways.

ROS is implicated in complex regulatory networks governing both biotic and abiotic stress responses [32, 33, 34] and also known to play role under combined stresses [35]. The ROS triggered downstream signaling events are also part of shared hormonal responses and metabolic pathways [36, 37]. These signalling networks interact as a part of ‘cross-talk’ and play role in plant adaptation to multiple individual stresses [38–41] and combined stress [10, 11, 17]. Apart from role in signaling pathway, high levels of ROS cause cellular damage due to oxidative stress. The antioxidant defense mechanism is one of the key pathways associated with number of individual and combined stresses [26, 42]. For example, under combined drought and heat stress tolerance, antioxidant enzyme cytosolic ascorbate peroxidase (APX1) plays critical role in H_2_O_2_ scavenging [43]. Besides several mutants defective in ROS scavenging enzymes showed increased susceptibility to both biotic and abiotic factors [34, 44, 45]. Catalase-deficient barley showed leaf bleaching [46] and tobacco CAT1 antisense lines showed necrotic lesions linked to the activation of certain pathogen responses [47,48]. In the recent past, many studies have used exogenous ROS generating chemical compounds and ROS scavenging systems as a potential tool to identify plants tolerant to multiple stresses [13, 49]. Genetic variability for oxidative stress tolerance in crop plants has also been explored to identify multiple stress tolerant crops [11, 44, 50, 51]. In our previous work we developed an empherical screening-based approach for identification of individual abiotic stress tolerant crops [50, 52, 53, 54, 55]. The selection criteria for tolerant seedlings during screening involved not only survival under stress after acclimation treatment, but also their high growth rate during recovery.

In the present study the major emphasis was to identify sunflower genotypes contrasting for oxidative stress tolerance using menadione, an oxidative stress inducer and understand the mechanisms involved in multiple individual and combined stress responses (S1 Fig). Initially, meta-analysis was performed on sunflower transcriptome datasets selected from six publically available biotic and abiotic stress experiments to identify commonly regulated genes with most up- and down-regulation. The analysis led to the identification of 526 up-regulated and 4440 down-regulated stress responsive genes which are shared across the stresses. Further RT-qPCR analysis of these genes confirmed their expression pattern observed in microarray. Subsequently expression pattern of the identified genes was studied using the sunflower genotypes having contrasting stress tolerance under multiple individual and combinations of stresses namely, drought, cold, methyl viologen, NaCl and pathogen. Results from this study revealed the plant responses to multiple individual and combined stresses and identified candidate genes for further studies on development of broad-spectrum stress-tolerant sunflower in the future.

## Materials and Methods

### Plant material and growth conditions

The sunflower genotypes were obtained from different centers of All India Coordinated Research Project (AICRP) for sunflower at University of Agricultural Sciences (UAS), GKVK Bangalore, India. The genetic backgrounds and agronomic characteristics of these 55 lines are described in S1. The sunflower seeds of var. Morden (an open pollinated heterogeneous population) were procured from National Seeds Project, UAS, GKVK, Bangalore, India. Two-day-old seedlings were grown on moist filter paper in Petriplates and incubated at 30°C in seed germination chamber. For the seedling level stress treatment, plants were grown in pots with 2 kg of soil under greenhouse conditions with 10/14 h day/night cycle, 27°C temperature and 80% relative humidity.

### Data collection and meta-analysis

The transcriptomic data of individual biotic and abiotic stresses on sunflower was collected from array express database (https://www.ebi.ac.uk/arrayexpress/) (S2 Table). This data (http://www.ebi.ac.uk/arrayexpress/experiments/browse.html?keywords=&organism=Helianthus+annuus&array) was manually curated using Microsoft Excel and control and treatment files were separated. The curated data were used as input files for meta-analysis. Integrative Meta-analysis of Expression data (INMEX) tool [56] was used for meta-analysis of multiple gene-expression datasets for identifying commonly up- and down-regulated genes. Stouffer’s model was used to integrate the data with treatments and controls, thereby commonly expressed (shared) genes among the different stress conditions were identified [57]. This method is used in meta-analysis of data across studies using p-value, sample size and estimated direction of effect for each study. This method can easily execute meta-analyses even when different analytical approaches were used in each individual study [58]. For the data upload, input data (file format.txt or.zip) was arranged in Excel file with gene expression values and corresponding probe ID or gene name in rows and samples or experiments in columns. Each column or treatment was named as per specific treatments. Further the up- and down-regulated gene IDs were converted from Affimetrix to Uniprot IDs. The different dataset were merged together into a mega-dataset (S2 Fig). The functional annotation of the identified genes was derived using Blast2GO tool [59]. Blast2GO identifies the function of a given sequence primarily based on the gene ontology (GO) term. It optimizes the function of a given sequence when compared to homologous sequences considering the similarity and the extent of homology in the selected database (https://www.blast2go.com/).

### Individual stress imposition

#### Oxidative stress in seedlings by menadione

Menadione (2-methyl-1, 4 napthoquinone) sodium bisulfite (Cat No. M2518-100G, Sigma Aldrich), a free radical inducer [50, 60] was used in this study to impose oxidative stress in seedlings 48 h after germination. Menadione is a quinone compound and upon auto oxidation and reduction process, it generates superoxide radicals in the cell. It is suitable for imposing oxidative stress in non-photosynthesizing tissues. Seedlings of 2.5–3 cm length were incubated at a particular concentration of menadione (0.25, 0.5, 1, 2, 3, 4, 5 mM) for 2 h at 30°C under constant shaking. After the treatment, seedlings were washed thoroughly using distilled water. A subset of the control seedlings were transferred to Petriplates on moist filter paper and allowed to recover for 3 d at 30°C. After the recovery period, survival and recovery growth were measured. Another subset of seedlings were allowed to recover for 5 h at 30°C and used to estimate cell death [50, 61, 62]. In all the experiments three replicates were taken and each replicate had 25 seedlings. The seedlings maintained at 30°C throughout the experimental period, were used as absolute controls. Reduction in growth of seedlings was calculated using the following formula.

$$\text{Reduction in growth over absolute control }\left(\text{\%}\right)=\frac{\text{Growth of seedlings after recovery}}{\text{Growth of seedlings in absolute control}}\text{ X}\;100$$

Ten-day-old sunflower seedlings were treated with 1 mM menadione and after 2 h the tissue from leaf, root and whole seedling were frozen. RNA from these tissues was extracted and cDNA was synthesized using the protocol described under RT-qPCR. Expression of oxidative stress responsive genes namely *superoxide dismutase* (*SOD*, accession number AY172569), *ascorbate peroxidase* (*APX*, accession number AGU36670), *catalase* (*CAT*, accession number L28740), and *heat shock protein* (*HSP17*, accession number U96641) were studied using RT-qPCR.

#### Oxidative stress in leaves by methyl viologen

Methyl viologen (Paraquat dichloride; M2254, Sigma Aldrich), a ROS generating herbicide in chloroplast was used to impose oxidative stress under 1400 μmol m^-2^s^-1^ in sunflower leaves. This compound interferes with photosynthetic electron transport chain to produce ROS in photosynthesizing tissues under high light. Oxidative stress was imposed by spraying 5 μM methyl viologen on 7-day-old plants and the tissue was collected after 12 h for different assays (S5 Fig).

#### Drought stress

Pots were filled with potting mixture of known weight and were irrigated until all the soil macro and micro pores were filled and excess water was drained overnight. Based on water holding capacity for this soil mixture total weight of pot with soil mix for 100% field capacity (FC) was arrived. Drought stress was imposed by gravimetric approach [63]. 10-day-old plants in pots were used for the experiment. Stress was imposed by withholding irrigation and the plants meant for drought stress were maintained at 30–40% FC for one week. At the end of stress period, stress responses were studied in the leaves.

#### NaCl stress

NaCl 200 mM was dissolved in water and irrigated to the pots having 7-day-old plants. After 5 days of treatment the leaf tissue was frozen for gene expression and biochemical studies (S5 Fig).

#### Cold stress

Plants (10-day-old) were subjected to cold stress by incubating at 4°C for 2 h and tissue was frozen for further studies (S5 Fig).

#### Downy mildew pathogen infection

Field grown plants (45-day-old) were naturally allowed to infect with *Plasmopara halstedii*. The uniformly infected symptomatic leaves were used to rub on the 7-day-old plants grown in pots. At the University of Agricultural Sciences downy mildew infected ‘sick plots’ are maintained for varietal trails [64]. The experiment was carried out during spring (humidity 60–70%, 28°C day and 16°C night temperature). In spring *P*. *halstedii* infects sunflower seedlings through germination of overwintered sexual oospores. For the systemic plant colonization by disseminating structures on various plant organs intercellular hyphae play critical role under humid conditions [65]. This pathogen causes seedling damping off, dwarfing of the plant, bleaching of leaves, and visible white sporulation on the lower side of leaves [65]. Disease index was scored after 5 days and tissue was collected for gene expression (S5 Fig). The pathogen infection incidence was assessed by scoring visible white spores and bleaching symptoms. Scoring was done as follows: 0 = no symptoms on the leaves; 1 = <1%; 2 = 1–10%; 3 = 10–25%; 4 = 25–50%; 5 = 50–75%; 6 = > 75% of total leaf area affected. Disease index (DI) was calculated using the following formula [66]:

$$\text{Disease index }\left(\text{DI}\right)=\frac{\text{Sum of numerical rating}\times 100}{\text{Total number of inoculated leaves }\times 6}$$

Six in the formula indicates maximum disease grade.

### Combined stress imposition

Ten-day-old plants were used for combined stress imposition. Two types of combined stresses were imposed in this study. One is combination of drought and pathogen, in which plants were initially exposed to drought stress by withholding the water for 3 days. The pathogen was inoculated on the first day of water withholding. The tissue was collected after 3 days of combined stress treatment. Second type of combined stress involved subjecting plants to combination of drought, NaCl, cold, oxidative and pathogen stress as per following procedure. Initially control grown plants were irrigated with 200 mM NaCl and then water was with-held for 3 days. During same period plants were simultaneously exposed to cold stress for 2 h and sprayed with 5 μm methyl viologen and inoculated with pathogen. All these process were carried out within 3 days period and tissue was frozen for further analysis. Minimum of three replicates were maintained for each treatments. The overview of the combined stress experiment is presented in S5 Fig.

### Estimation of H_2_O_2_

The levels of H_2_0_2_ play critical role in signaling and act as substrate for reactive oxygen species (ROS) [67] and we quantified the stress induced H_2_0_2_ using xylenol orange assay [68]. The xylenol orange reagent was prepared in 50 ml of distilled water containing 1 mL of 50 mM ferrous ammonium sulphate in 2.5 M H_2_SO_4_ and 62.5 μL of 125 μM xylenol orange (Sigma chemicals, cat No. 52097-5G, Bangalore) and 0.9019 g sorbitol. The tissue sample was extracted in phosphate buffer (pH 7.5). From this 25 μL supernatant was taken and mixed with 275 μL of xylenol orange reagent. The reaction mix was incubated for 30 min at room temperature and absorbance was measured at 560 nm against xylenol orange reagent only as blank [68]. Standards were prepared by dilution of reagent grade 30% H_2_O_2_.

### Estimation of Melandialdehyde (MDA) content

Melandialdehyde (MDA) is the end product of lipid peroxidation. MDA levels are indicators of extent of stress impact on plant cell membrane. Leaf tissue (1.0 g) was homogenized in 5 mL of 5% (w/v) trichloroacetic acid and the homogenate was centrifuged at 12,000 g for 15 min at room temperature. The supernatant was mixed with an equal volume of thiobarbituric acid [0.5% in 20% (w/v) trichloroacetic acid], and the mixture was boiled for 25 min at 100°C, followed by centrifugation for 5 min at 7,500 g to get clear solution. Absorbance of the supernatant was measured at 532 nm. MDA content in leaf tissue was calculated using standard graph developed using MDA (Sigma chemicals cat No.63287-1G-F, Bangalore) [69].

### Real-time quantitative RT-PCR (RT-qPCR)

Total RNA was extracted according to the protocol described by Datta et al. [70]. First strand cDNA was synthesized by oligo (18 mer dT) primers using *Molony murine leukaemia virus* reverse transcriptase (MMLV-RT; MBI Fermentas, Hanover, MD, USA) according to manufacturer’s instructions. The cDNA pool was used as a template to perform RT-qPCR analysis. PCR reactions were performed in optical 96-well plates (Applied Biosystems) with an ABI PRISM^®^ 7900 HT sequence detection system, using SYBR^®^ Green to monitor the synthesis of double-stranded DNA. A standard thermal profile with the following conditions was used, 50°C for 2 min, 95°C for 10 min, 40 cycles of 95°C for 15 s, and 60°C for 1 min. Amplicon dissociation curves were recorded after cycle 40 by heating from 60 to 95°C with a ramp speed of 1.9°C min^−1^. The relative expression levels of the selected genes under a given stress condition was calculated using comparative threshold method by comparing reference control gene [71]. *Actin* (FJ487620.1) and *Ubiquitin* (X14333.1) were used as internal controls to normalize RT-qPCR. Details of all primers used in this study are given in S3 Table.

### Statistical analysis

The data obtained was analysed using two-way analysis of variance (ANOVA) as per the procedure given by Fischer [72]. Data points with ‘*’ indicate significant differences at P≤0.05.

## Results

### Identification of commonly regulated genes under abiotic and biotic stresses using meta-analysis of transcriptome data

The sunflower cDNA arrays used in this study were derived from transcriptomic studies available from the public databases. The data from plants exposed to drought, heat, NaCl, oxidative stress, cold stress and an oomycete pathogen, *Plasmopara halstedii* (causal agent of downy mildew in sunflower) infection were collected to identify stress responsive genes shared among these stresses (S2 Table).

**Fig 1:**
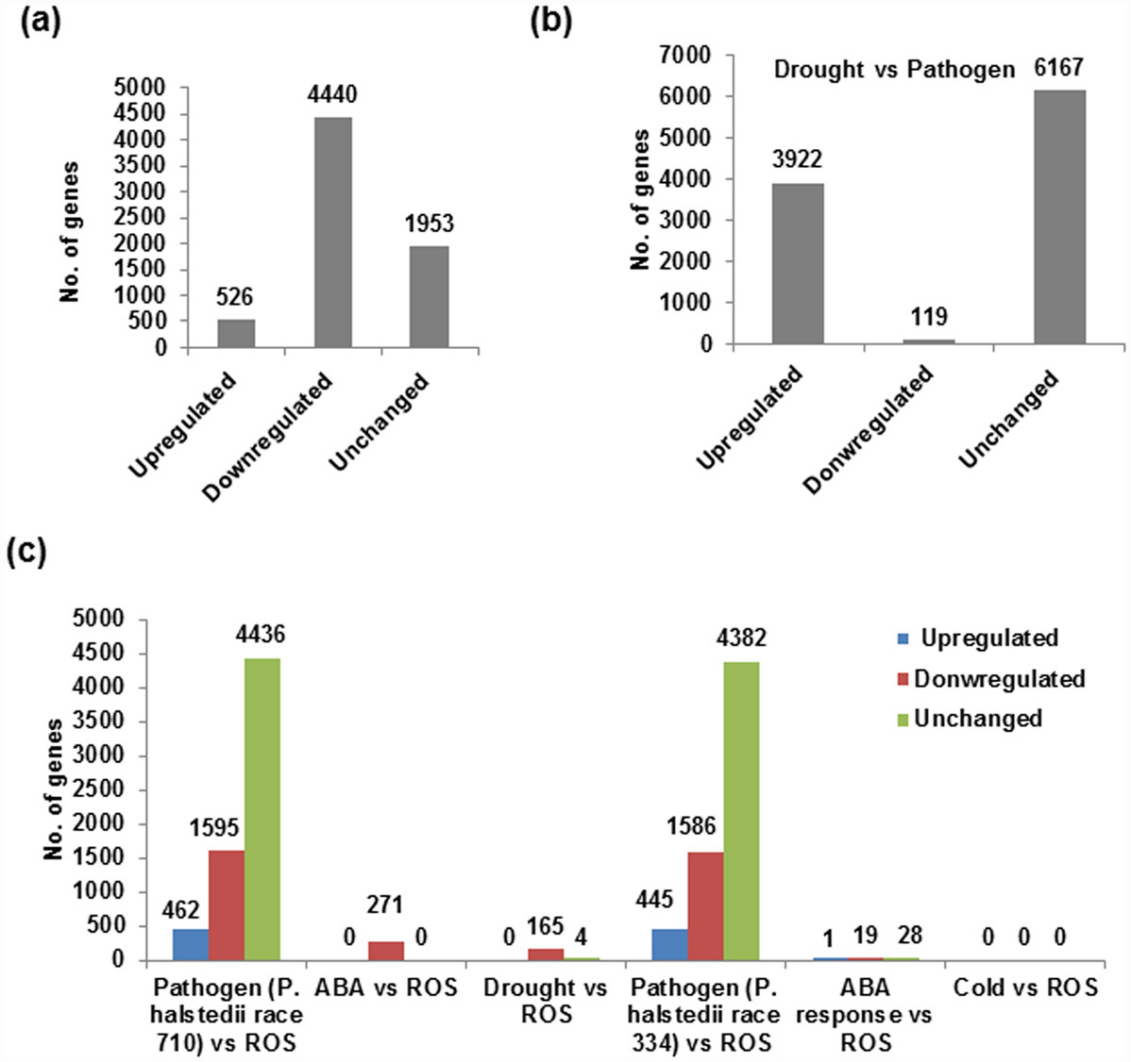
Meta-analysis of sunflower transcriptome data from 6 different experimental datasets. The raw data were integrated in meta-analysis tool INMEX and differentially expressed genes were identified. The number of differentially expressed genes under all stresses (a), between drought and pathogen stresses (b). Based on individual stress comparisons, commonly up-regulated, down regulated and unchanged genes were identified (c).

To identify the commonly up or down-regulated genes across the six stresses, meta-analysis was performed. The overall experimental approach followed is detailed in S2 Fig. The analysis showed 526 up-regulated, 4440 down-regulated genes and 1953 genes with similar expression like control (Fig 1). The number of genes upregulated in drought and pathogen was higher than all other stresses. Analysis of differentially expressed genes specifically under drought and pathogen stress showed 3922 up-regulated and 119 down-regulated genes. This data indicated that several genes are shared under multiple individual stresses (Fig 1b). The analysis showed no genes shared between cold and oxidative stress (Fig 1c). On the contrary maximum number of shared genes were found between pathogen stresses (two races of downy mildew pathogen) and oxidative stress. Particularly, 1595 and 1586 genes were down-regulated and 462 and 445 genes were up-regulated in race 710 and race 334, respectively. Further, ABA-ROS and drought-ROS comparison also revealed several commonly regulated genes.

The up-regulated genes shared across the stress were classified into different classes based on their molecular function using Blast2GO tool. Large number of genes were found to be involved in protein binding (9.8%), ATP binding function (7.3%), oxidoreductases (4.5%), DNA and RNA binding (4.2 & 4%) in addition to hydrolases, ligases, zinc ion binding, kinase activity, transcription factors and membrane transporters (S6 Fig). The remaining genes had unknown function. Further 29 genes commonly up- or down-regulated in many stresses were directly or indirectly involved in regulation of ROS and oxidative stress tolerance were shortlisted for further analysis (S4 Table). The upregulated genes were subjected to Agrigo tool to map the genes to identify associated pathways and based on biological function, those genes were found to be involved in oxidation reduction process (S7 Fig). Similarly in the large number of down regulated genes were involved in developmental processes, hormone responses, defense responses, transcription, translational events and protein modifications (S8 Fig). The genes involved in plant development, including anatomical structure (23 genes), flower (8 genes), pollen (6 genes) and seed (8 genes) development responsive genes were downregulated. The downregulated genes with response to stimulus include multicellular organismal process (34 genes), responses to stress (34 genes), responses to chemical stimulus (28 genes), abiotic stimulus (19 genes) and defense responsive (14 genes). The genes that were involved in many biosynthetic process (57 genes), macromolecular biological process (82 genes), catabolic process (16 genes), macromolecule modification (25 genes), protein modification (24 genes) and post translational modification (20 genes) were downregulated. Based on these results and literature information [73] we hypothesised that oxidative stress tolerance mechanisms are linked to tolerance of plants to multiple number of individual stresses and also combined stresses.

### Menadione induces oxidative stress and broad-spectrum stress effects

Menadione, a compound that produces superoxide radicals, has been used to induce oxidative stress in plants [50, 74, 75]. Seedlings (two day old) of var. Morden were treated with different concentrations of menadione and response was recorded after recovery. Mild concentrations of menadione (0.25 to 2 mM for 2 h) reduced the shoot and root growth compared to that of water treated controls. Root and shoot growth was reduced at concentration higher than 3 mM menadione treatment for 2 h (S9 Fig). Seedling survival and growth after recovery period were reduced as the concentration of menadione and duration of incubation increased. However at high concentrations of menadione (LD_50_ = 2 mM-3 h or 3 mM-0.5 h), the seedlings abruptly collapsed due to the cell death as quantified by Evans blue staining (S9 Fig; S5 Table).

**Fig 2:**
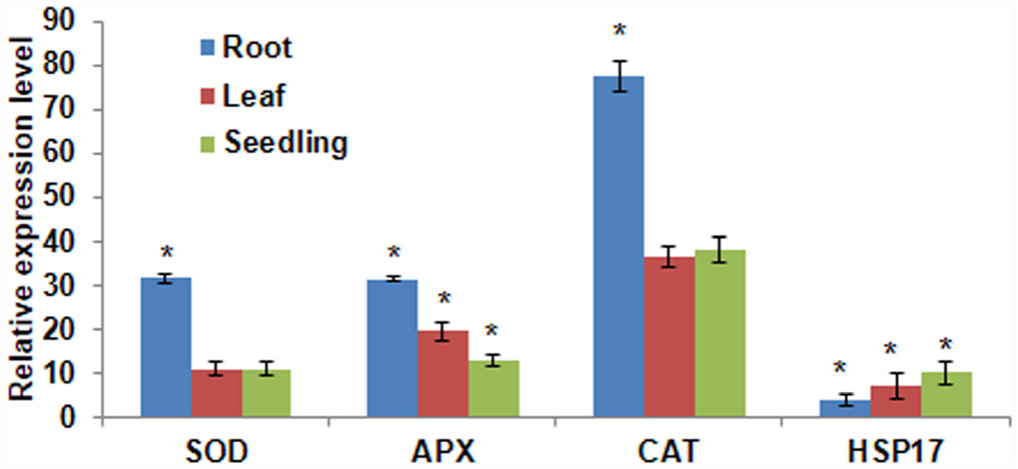
Transcript expression profile of oxidative stress responsive genes in menadione treated sunflower seedlings. Expression pattern of *HaSOD*, *HaAPX*, *HaCAT*, *and HaHSP17* were studied using RT-qPCR. Sunflower seedlings were exposed to 1 mM menadione for 2 h. RNA was extracted after the stress period from root, leaf and whole seedling and cDNA was synthesized. ‘*’asterisks indicate a significant difference from the control (two way ANOVA and Duncan’s multiple range test at P<0.05). Error bars indicate standard error of mean of three biological replicates.

Further the expression of few known stress responsive genes was tested in the menadione stressed seedlings (var. Morden). The expression levels for *SOD*, *APX*, *CAT* and *HSP* genes were up-regulated compared to non-stress seedlings in roots and leaf. The transcript expression of these genes in seedlings was similar to the expression in roots (Fig 2). This indicated that menadione-induced oxidative stress enhances the expression of genes involved in ROS scavenging and stress adaptation.

### Menadione stress screening identifies genotypes contrasting in stress tolerance

**Fig 3:**
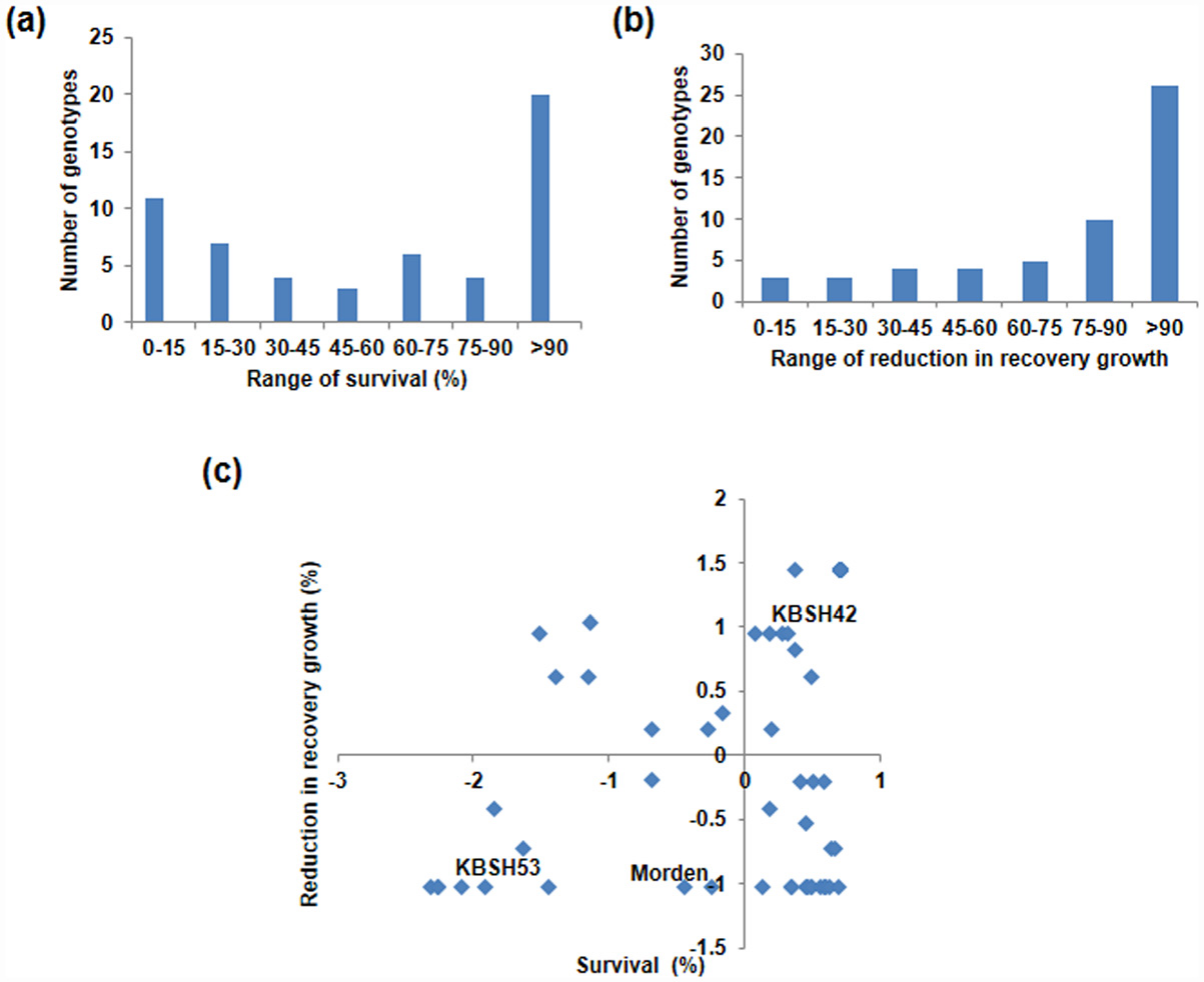
Genetic variability of sunflower genotypes under menadione-induced oxidative stress. Frequency distribution of genotypes based on survival (%) (a) and, reduction in recovery growth (%) (b) and seedlings were classified using Z- distribution analysis for the 55 genotypes (c). The first and fourth quadrant indicates susceptible and resistant genotypes respectively. The seedlings were exposed to acclimation stress of 1 mM menadione at 30°C and subsequently exposed to a higher concentration of menadione.

To identify the contrasting genotypes, seedlings of 55 sunflower genotypes were subjected to menadione stress and survival and recovery growth were recorded (Fig 3a & 3b). During the national trials, these genotypes were grown in various geographical locations in India including Akola, Bangalore, Coimbatore, Dholi, Hisar, Ludhiana, Nandyal, Nimpith and Raichur. Owing to the characteristic abiotic stress occurrence in these locations, they were exposed to various stresses during their growth season. Six genotypes were found to be extremely sensitive to oxidative stress based on survival and recovery data (S6 Table). The Z-distribution analysis for both survival and recovery growth was used to identify contrasting genotypes. Based on this, two contrasting genotypes namely KBSH53 and KBSH42 were identified as tolerant and susceptible genotypes, respectively (Fig 3c). These two genotypes were also contrast for resistance to powdery mildew (S1 Table). These two genotypes and another variety Morden identified through temperature induced stress response is moderately tolerant and high yielding, were used to study the effect of multiple individual and combined stresses.

### Oxidative stress tolerant genotypes exhibits tolerance to multiple individual and combined stresses

**Fig 4:**
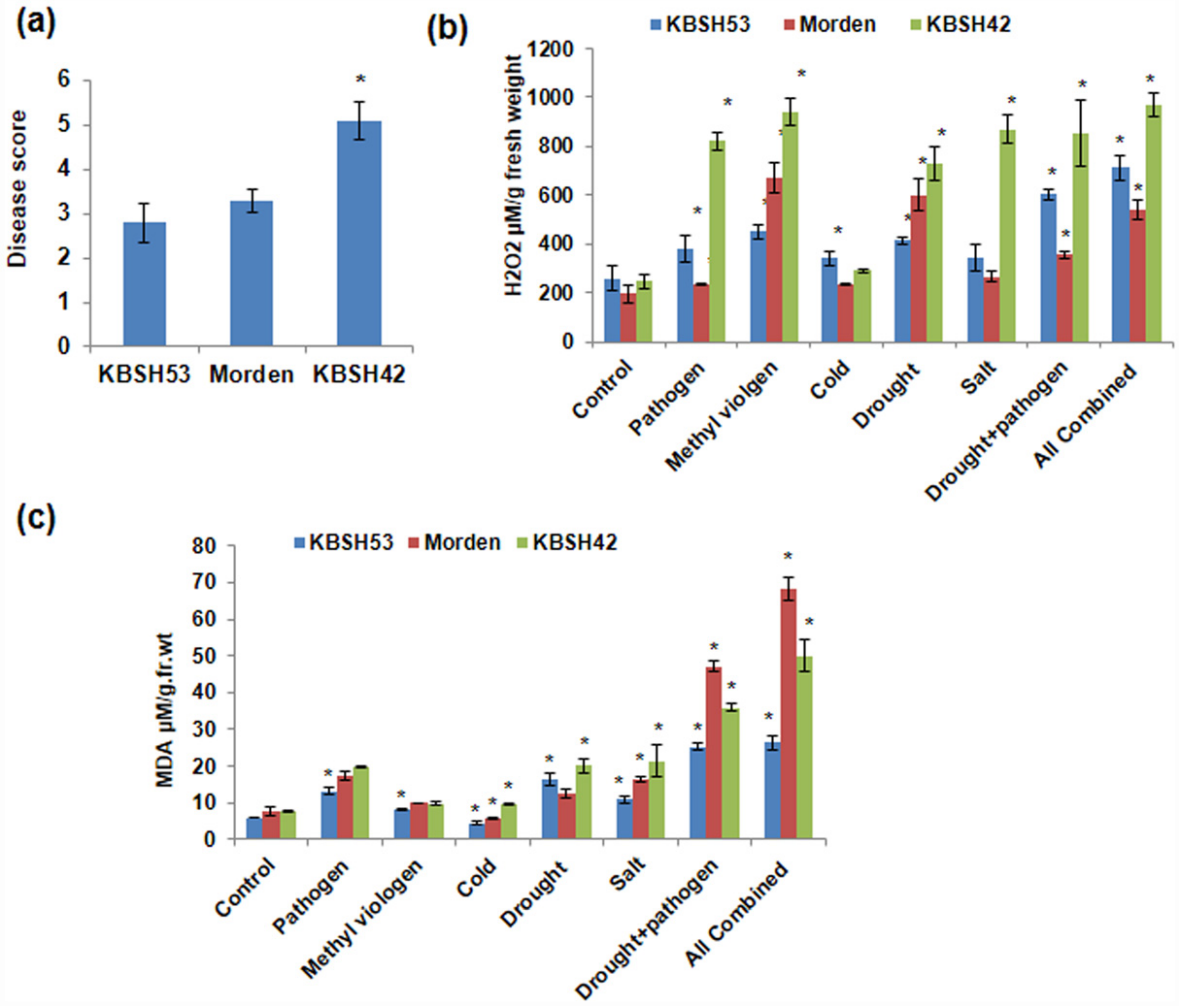
Individual and combined stress response of the three genotypes varying in oxidative stress tolerance. Disease score on plants exposed to *P*. *halstedii* (a) 7-day-old sunflower plants were exposed to pathogen spores for 5 days and. Score was assigned from 1–10 based on low to high infection. H_2_O_2_ levels were assessed from leaves of different biotic and abiotic stressed plants (b). The leaf tissue was ground in PBS buffer and aliquots were used for estimation of H_2_O_2_ levels by using a modified ferrous oxidation-xylenol orange (FOX) assay. MDA levels in stressed plants (c) was quantified by TBARS assay to study the extent of damage on lipids. ‘*’ indicate a significant difference from the control (Student’s t test, P<0.05). Error bars indicate standard error of mean. Data were pooled from two independent experiments representing three biological replicates.

To test tolerant genotypes identified through menadione-based screening for their response to multiple individual and combined abiotic and biotic stresses, these genotypes were subjected to different stresses as shown in the S5 Fig. KBSH53 showed less disease index as compared to KBSH42 (Fig 4a). In these genotypes accumulation of ROS under drought plus pathogen combined stress and all (pathogen, NaCl, drought, cold and methyl viologen) combined stresses is significantly higher than the independent stresses as shown by NBT staining (S10 Fig). KBSH53 showed less NBT staining under pathogen and other treatments and KBSH42 showed higher accumulation of superoxide radicle and higher accumulation of H_2_O_2_ as compared to KBSH53 in all stresses (Fig 4b & S10 Fig). Similarly, MDA levels showing lipid peroxidation was high in KBSH42 and low in resistant genotype KBSH53 (Fig 4c). Consistently, susceptible genotype KBSH42 showed higher lipid peroxidation. This confirms that the genotypes identified through menadione screening showed response similar to that exhibited under oxidative stress under multiple individual and combined drought and pathogen stress. Taken together, large scale screening of sunflower genotypes using menadione identified contrasting genotypes KBSH42 and KBSH53 for individual and combined stress tolerance (Fig 4).

### Gene expression analysis under individual and combined stress explains the molecular basis for susceptibility and resistance of genotypes

**Fig 5:**
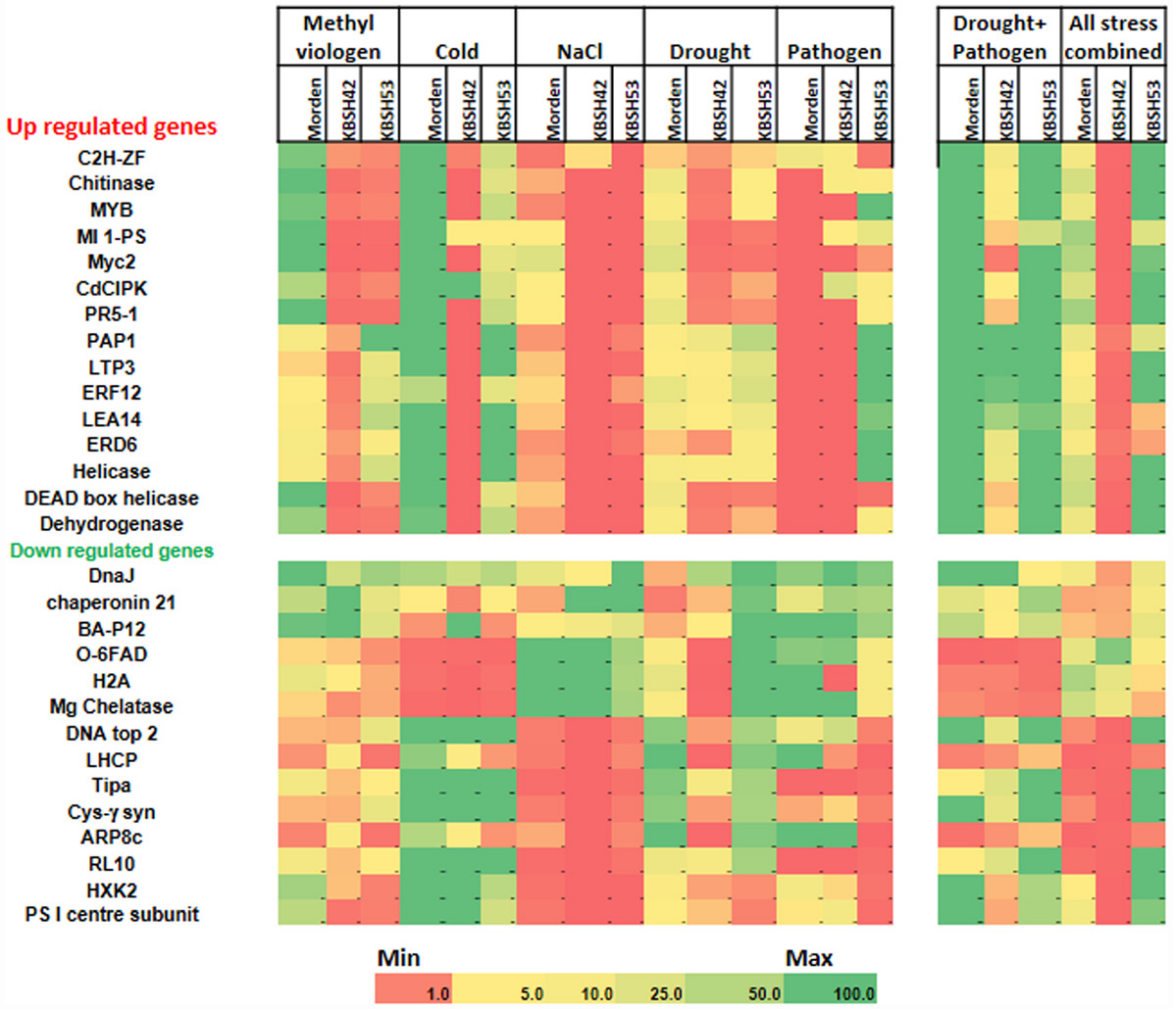
Transcript profiling of sunflower genotypes varying in stress tolerance under individual and combined stresses. Morden, KBSH42 and KBSH53 plants were subjected to individual stresses namely, methyl viologen-induced oxidative stress, cold, salt, drought and pathogen. Another batch of plants were subjected to two type of combined stresses, namely drought and pathogen and all stresses combined. The stress protocol is described in S5 Fig. From these stressed plants total RNA was isolated and cDNA was prepared and used for RT-qPCR. Three replicates were maintained. The expression was normalized to *HaActin* and fold change was calculated against the control samples. Two way ANOVA and Duncan’s multiple range test at P<0.05 was carried out using three biological replicates.

To study the gene expression pattern in contrasting genotypes under individual and combined stresses, a total of 15 up- and 14 down-regulated genes that were selected from meta-analysis were used for RT-qPCR (S4 Table). The transcript analysis showed that many genes identified by meta-analysis were up-regulated in the tolerant genotype KBSH53 in all combined stresses and drought plus pathogen stress. The transcript levels were higher for genes encoding DNA topisomerase, aquoglyceroporin, cystathionine **γ**-synthase, envelope glycoprotein RL10, hexokinase, and photosystem I reaction center proteins under both type (drought plus pathogen, and pathogen, NaCl, drought, cold and methyl viologen all together) of combined stresses. However, the majority of the transcripts upregulated either one fold or less in individual stresses. Many genes upregulated in meta-analysis data also upregulated in the drought plus pathogen combined stress (Fig 5).

In the susceptible genotype KBSH42 several genes showed reduced transcript levels under individual and combined drought plus pathogen stresses. Only *Microsomal oleic acid desaturase* (*O-6FAD*) gene showed up-regulation in all combined stress. The *DnaJ* gene found to be down-regulated in meta-analysis results showed up regulation under combined drought plus pathogen stresses (Fig 5). Similarly, the genes encoding *acid phosphatase 1*, *lipid transfer protein isoform 3*, *ethylene responsive transcription factor 3* and *late embryogenesis abundant 4* were up-regulated in combined drought plus pathogen stress which showed similar trend of transcript levels as predicted by meta-analysis. In nutshell, the transcript profiling of selected genes both under individual and combined stresses showed that the transcriptome response in KBSH53 is different from KBSH42 (Fig 5).

## Discussion

### Meta-analysis is a useful tool to identify shared genes among multiple individual and combined stresses

Understanding the shared mechanisms contributing to two or more individually or simultaneously occurring stresses is important to improve crop productivity under foreseeable complex stress situations. But, adaptation of plants to such individual and combined stress is imparted through a complex, yet to be fully understood mechanisms. To dissect molecular mechanism behind multiple individual stress and combined stress tolerance in sunflower, meta-analysis approach was employed [29] using publically available transcriptome datasets from individual stress studies. The analysis revealed 526 genes up-regulated and 4440 genes down-regulated among *P*. *halstedii* infection, ROS treatment, drought, ABA treatment and cold stresses. Most of the commonly up- or down- regulated genes identified from meta-analysis showed similar expression pattern under all combined stresses. Between ROS and cold stress response no commonly regulated genes were found. A simple explanation is either the genes responsive to cold and ROS are independent or the levels of stress imposed was not sufficient to trigger the shared responsive genes. Role of several of these genes under multiple individual and combined stresses are largely unknown (S4 Table). The genes encoding C2H2 zinc finger, MYB, MYC/bHLH and ethylene responsive factor (ERF) belong to specific family of transcription factors. These transcription factors are known to regulate several downstream functional genes in response to different environmental stresses [9, 11]. Another interesting class of genes found were those encoding H2A, DNA topoisomerase 2, DNaJ and DEAD box helicases. Apart from these, genes involved in histone relaxation, DNA repair and RNA secondary structure removal under stress were also found [76, 77, 78, 79]. Further the downstream genes encoding chitinase, pathogen resistance 5 (PR5), autophagy related protein and myo-inositol-1-phosphate synthase that are involved in plant defense against pathogens were identified along with several chlorophyll and light harvesting complex protein encoding genes. A similar group of genes identified through microarray profiling of sunflower leaves exposed to cold and NaCl stress showed dynamic changes in transcript levels of transcription factors, genes related to translation, protein degradation/folding and ROS production or scavenging mechanisms [80]. Similarly comparative gene expression analysis under highlight (HL), high temperature (HT) and combined HL and HT stresses in sunflower leaves and seeds revealed differential expression of 89, 113 and 186 genes, respectively [81]. Meta-analysis of 6 experimental datasets under different stresses revealed several genes belonging to ATP-, DNA-, RNA-, protein- binding, hydrolase, ligase, oxidoreductase, serine threonine kinase, transcription factor, zinc ion binding and transporter activity. The data suggests that meta-analysis approach can be potentially employed to identify shared stress responsive genes, which can reveal the mechanism of combined and multiple stress tolerance.

Owing to the complexity involved in handling all combined stresses, we further focused on pathogen and drought combination for detailed systematic confirmation of the meta-analysis results and to dissect the shared mechanism between individual and combined stresses. Interestingly the meta-analysis showed that a large number of commonly regulated genes belong to the ROS-responsive or oxidative stress scavenging system (S7 Fig). ROS scavenging proteins are shown to act as early sensors to prevent potential oxidative stress damage [82]. The response of 187 nuclear encoded ROS responsive genes and 1880 transcription factors showed rapid and coordinated expression under H_2_O_2_ [83]. This prompted us to further examine the role of ROS pathway related genes using RT-qPCR under combined stress. Under combined drought and pathogen stress, the genes identified as up-regulated by meta-analysis consistently showed higher transcript levels in var. Morden. Interestingly most of the genes identified from the analysis were also induced in sunflower seedlings treated with menadione-induced oxidative stress (Fig 5). Since these genes were also separately confirmed for their up-regulation under combined drought and pathogen stress, we speculated the strong overlap in some gene expression between the methyl viologen or menadione-induced oxidative stress and the combined stress. This overlap can be attributed as shared response of plants among the combined and oxidative stresses. Meta-analysis of drought, bacterial stress response in rice and *A*. *thaliana* revealed 38.5% (1214) and 28.7% (272) differentially expressed genes (DEGs) respectively and a majority of these showed conserved expression status in both stresses (30). These studies suggests that several genes act as part of shared response between combined and individual stresses.

### Tolerant genotypes identified through menadione-based screen showed multiple individual and combined stress tolerance

We hypothesized that menadione-based screening of genetically diverse sunflower genotypes could identify tolerant and susceptible groups not only for oxidative stress tolerance, but also for tolerance to combined stresses (S5 Fig). Specifically, menadione-based screen has been earlier demonstrated as one of the methods suitable for screening and identification of contrasting stress tolerant genotypes in sunflower [50]. Moreover, owing to highly cross- pollinated nature of sunflower, the selected 55 genotypes are expected to have genetic variability for multiple individual and combined stress tolerance. The pool of genotypes used in this study represent genetic background with tolerance to various abiotic stresses, namely drought, temperature extremes and salinity apart from superior agronomical characteristics (S1 Table). Our screening process identified KBSH53 and KBSH42 as tolerant and susceptible genotypes, respectively. Interestingly, the susceptible genotype also showed susceptibility to combined drought and pathogen stress (Fig 4). Consistently, the resistant genotype showed improved performance under individual drought and pathogen stress and also combined stress. Taken together, these evidences support our hypothesis and show that the approach used in this study can identify not only genes responsible for multiple individual stress tolerance, but also for the combined stress. The usefulness of data from individual stress studies to identify genes for combined stress tolerance is possible because of crosstalk between many signalling pathways during multiple stresses [32, 33, 84, 85].

### Meta-analysis identified genes in multiple individual and combined stress tolerance

Meta-analysis of sunflower transcriptome datasets revealed 526 up and 4440 down-regulated genes in all combined stresses. RT-qPCR results for selected 29 genes in the tolerant genotype KBSH53 revealed candidate genes for combined stress tolerance. Amongst these genes, 17 were induced under all combined stress (pathogen, NaCl, drought, cold and methyl viologen stress) in tolerant genotype KBSH53, but susceptible genotype did not show transcript changes over control. In general, under any of the independent stresses the identified genes did not show significant fold change in both up- or down-regulated gene category. Overall the data suggest that under combined or multiple stresses, the meta-analysis can identify candidate shared stress responsive genes.

### Cross-talk and role for identified genes

Several genes showed up-regulation in the tolerant genotype KBSH53 including increased transcript levels of transcription factors C2H-ZF, MYB, MYC2, ERF12 and ERD6. Overexpression of some of these transcription factors resulted in multiple stress tolerance [86]. Since the literature information on validation is scarce, many other genes identified in the meta-analysis could not be verified for their functional relevance. However, we subsequently review few other evidences that support correlation between meta-analysis identified genes and their validation in literature. The induction of transcription factors under combined stress has been reported in a previous study, wherein it was observed that combined heat and drought stress lead to upregulation of WRKYs and ERFs [87]. One of our previous study also showed the overexpression of *AtWRKY28* in *A*. *thaliana* enhances drought and NaCl stress tolerance [88, 89]. The tolerant genotype also showed up-regulation of transcriptional regulators such as DNA topoisomerase 6, DEAD box helicases, ribosomal protein L10 (RPL10), ROS detoxification enzyme encoding genes like dehydrogenases, genes involved in protein stability such as chaperonins, late embryogenesis abundant 14 (LEA14), myo inositol phosphate synthase, calcium induced protein kinase (CIPK), lipid transport proteins and histidine kinases. It is evident that under combined stress, receptor like kinases, protein kinases (MAPK and CIPK), small GTP- binding proteins and membrane intrinsic proteins (MIP) are up-regulated [90]. Further, PR and chitinases also showed up-regulation in tolerant genotype and these genes are independently known to impart resistance to different pathogens [84]. These genes up-regulated under combined stress have potential to improve stress tolerance through complex network mode of mechanisms. Taken together, our data demonstrated that the meta-analysis can efficiently identify the potential candidate genes for combined stress tolerance.

In conclusion, salient features of this study include, one, menadione-based screening can be used as means to generate oxidative stress and explore genetic variability in agronomically superior genotypes for oxidative stress tolerance. Second, meta-analysis can be potentially employed to identify candidate genes for multiple and combined stress tolerance. Third, identified genes are the potential candidates for genetic engineering of plants to combat multiple environmental stresses.

## Supporting Information

S1 Fig Overview of experimental plan and hypothesis tested in this study. (PPTX)

S2 Fig Flow chart depicting stepwise protocol followed to identify the shared genes in different individually occurring biotic and abiotic stresses. (PPTX)

S3 Fig General protocol to study the acclimation response of sunflower seedlings and plants. (PPT)

S4 Fig Protocol for screening of genotypes using menadione-induced oxidative stress. (PPTX)

S5 Fig Protocol for individual and combined stress imposition in sunflower. (PPTX)

S6 Fig Classification of upregulated genes identified from meta-analysis based on molecular function using Blast2go tool. (PPTX)

S7 Fig Mapping of upregulated genes based on biological functions. (PDF)

S8 Fig Mapping of downregulated genes based on biological functions. (PDF)

S9 Fig Effect of different concentrations of menadione on sunflower seedlings. (PPTX)

S10 Fig Superoxide radical quantification by NBT staining. (PPTX)

S1 Table Genetic background, agronomic characteristics testing locations of sunflower varieties and hybrids used for screening in this study. (DOCX)

S2 Table Details of sunflower material used, stress treatments and microarray source used in the transcriptomic data downloaded from public sources. (DOCX)

S3 Table List of genes and primer sequences used in the study. (DOCX)

S4 Table List of genes identified from meta-analysis which are shared across multiple individual stresses. (DOCX)

S5 Table Effect of different concentrations of menadione on sunflower seedling survival and recovery growth. (DOCX)

S6 Table Response of sunflower genotypes to menadione induced oxidative stress. (DOCX)
