# Supplementary material for: Transcriptome Analysis of Sunflower Genotypes with Contrasting Oxidative Stress Tolerance Reveals Individual- and Combined- Biotic and Abiotic Stress Tolerance Mechanisms
Source: PLoS One. 2016 Jun 17;11(6):e0157522. doi: 10.1371/journal.pone.0157522 (PMC4912118; doi:10.1371/journal.pone.0157522)
Supplement: S1 Fig — (PPTX) [file pone.0157522.s001.pptx]

## Slide 1
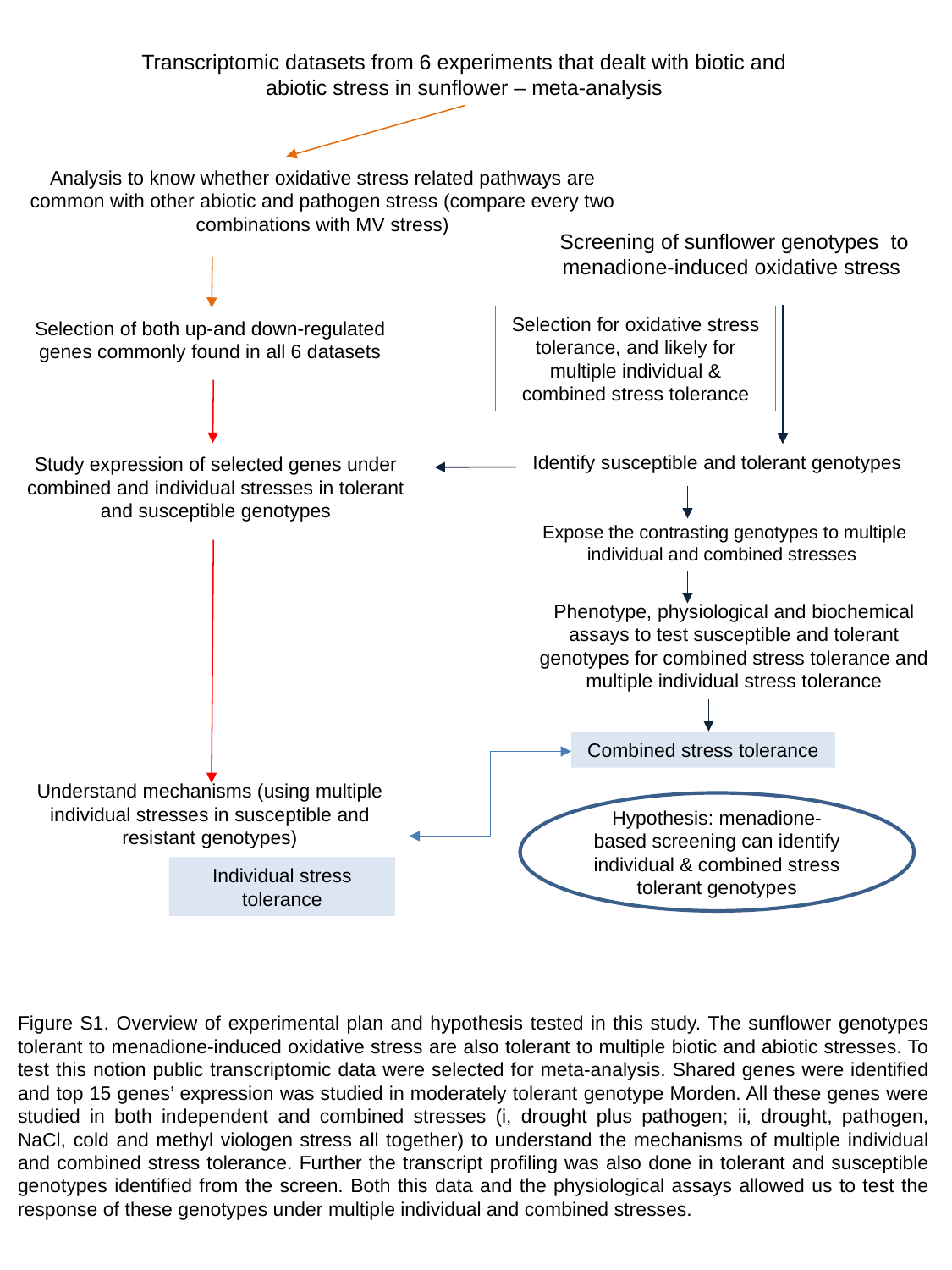

Transcriptomic datasets from 6 experiments that dealt with biotic and abiotic stress in sunflower – meta-analysis
Analysis to know whether oxidative stress related pathways are common with other abiotic and pathogen stress (compare every two combinations with MV stress)
Screening of sunflower genotypes to menadione-induced oxidative stress
Selection for oxidative stress tolerance, and likely for multiple individual & combined stress tolerance
Selection of both up-and down-regulated genes commonly found in all 6 datasets
Identify susceptible and tolerant genotypes
Study expression of selected genes under combined and individual stresses in tolerant and susceptible genotypes
Expose the contrasting genotypes to multiple individual and combined stresses
Phenotype, physiological and biochemical assays to test susceptible and tolerant genotypes for combined stress tolerance and multiple individual stress tolerance
Combined stress tolerance
Understand mechanisms (using multiple individual stresses in susceptible and resistant genotypes)
Hypothesis: menadione-based screening can identify individual & combined stress tolerant genotypes
Individual stress tolerance
Figure S1. Overview of experimental plan and hypothesis tested in this study. The sunflower genotypes tolerant to menadione-induced oxidative stress are also tolerant to multiple biotic and abiotic stresses. To test this notion public transcriptomic data were selected for meta-analysis. Shared genes were identified and top 15 genes’ expression was studied in moderately tolerant genotype Morden. All these genes were studied in both independent and combined stresses (i, drought plus pathogen; ii, drought, pathogen, NaCl, cold and methyl viologen stress all together) to understand the mechanisms of multiple individual and combined stress tolerance. Further the transcript profiling was also done in tolerant and susceptible genotypes identified from the screen. Both this data and the physiological assays allowed us to test the response of these genotypes under multiple individual and combined stresses.
