# Supplementary material for: Transcriptome Analysis of Sunflower Genotypes with Contrasting Oxidative Stress Tolerance Reveals Individual- and Combined- Biotic and Abiotic Stress Tolerance Mechanisms
Source: PLoS One. 2016 Jun 17;11(6):e0157522. doi: 10.1371/journal.pone.0157522 (PMC4912118; doi:10.1371/journal.pone.0157522)
Supplement: S2 Fig — (PPTX) [file pone.0157522.s002.pptx]

## Slide 1
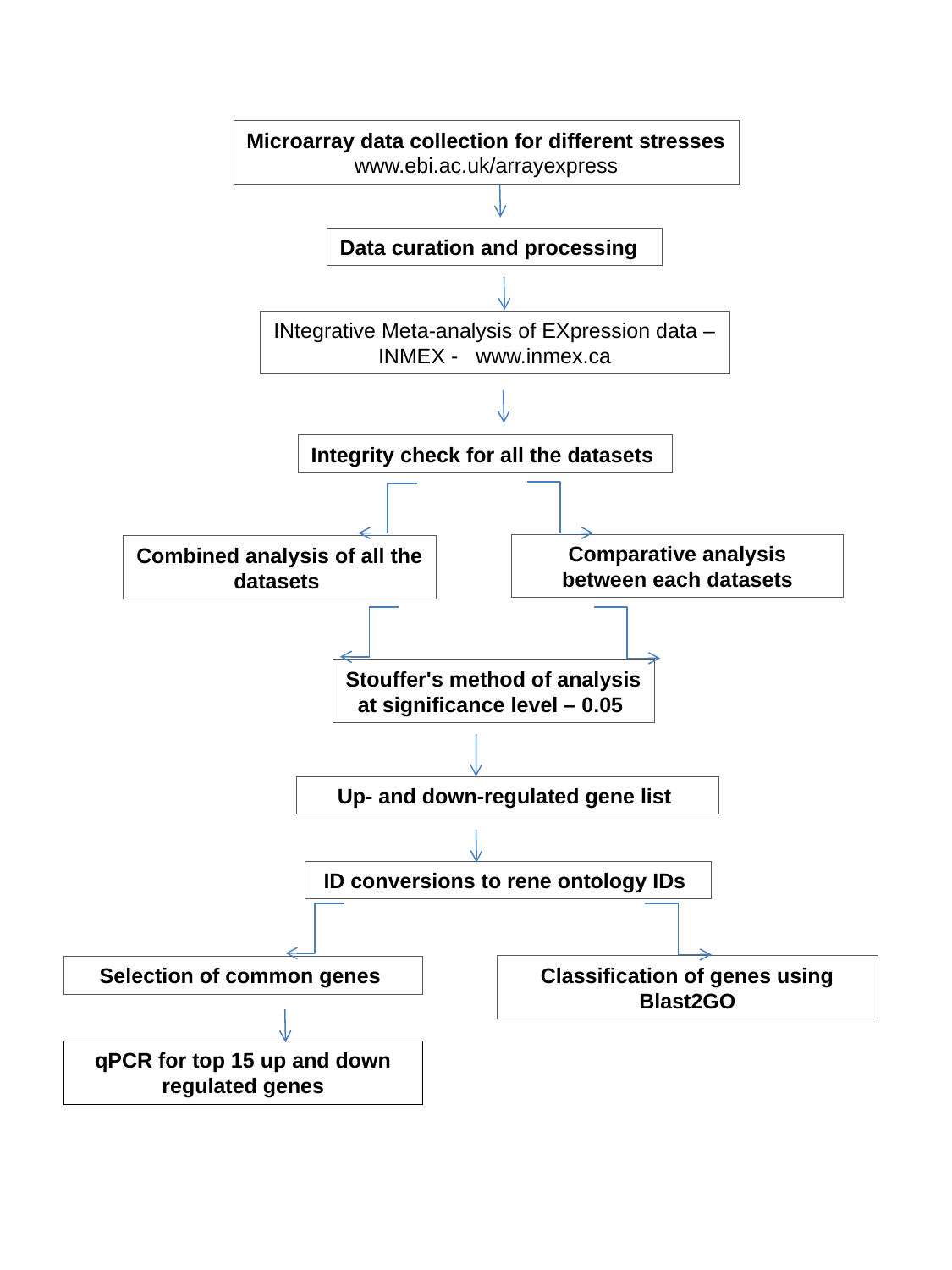

Microarray data collection for different stresses
www.ebi.ac.uk/arrayexpress
Data curation and processing
INtegrative Meta-analysis of EXpression data –INMEX - www.inmex.ca
Integrity check for all the datasets
Comparative analysis between each datasets
Combined analysis of all the datasets
Stouffer's method of analysis
 at significance level – 0.05
Up- and down-regulated gene list
ID conversions to rene ontology IDs
Classification of genes using Blast2GO
Selection of common genes
qPCR for top 15 up and down regulated genes

## Slide 2
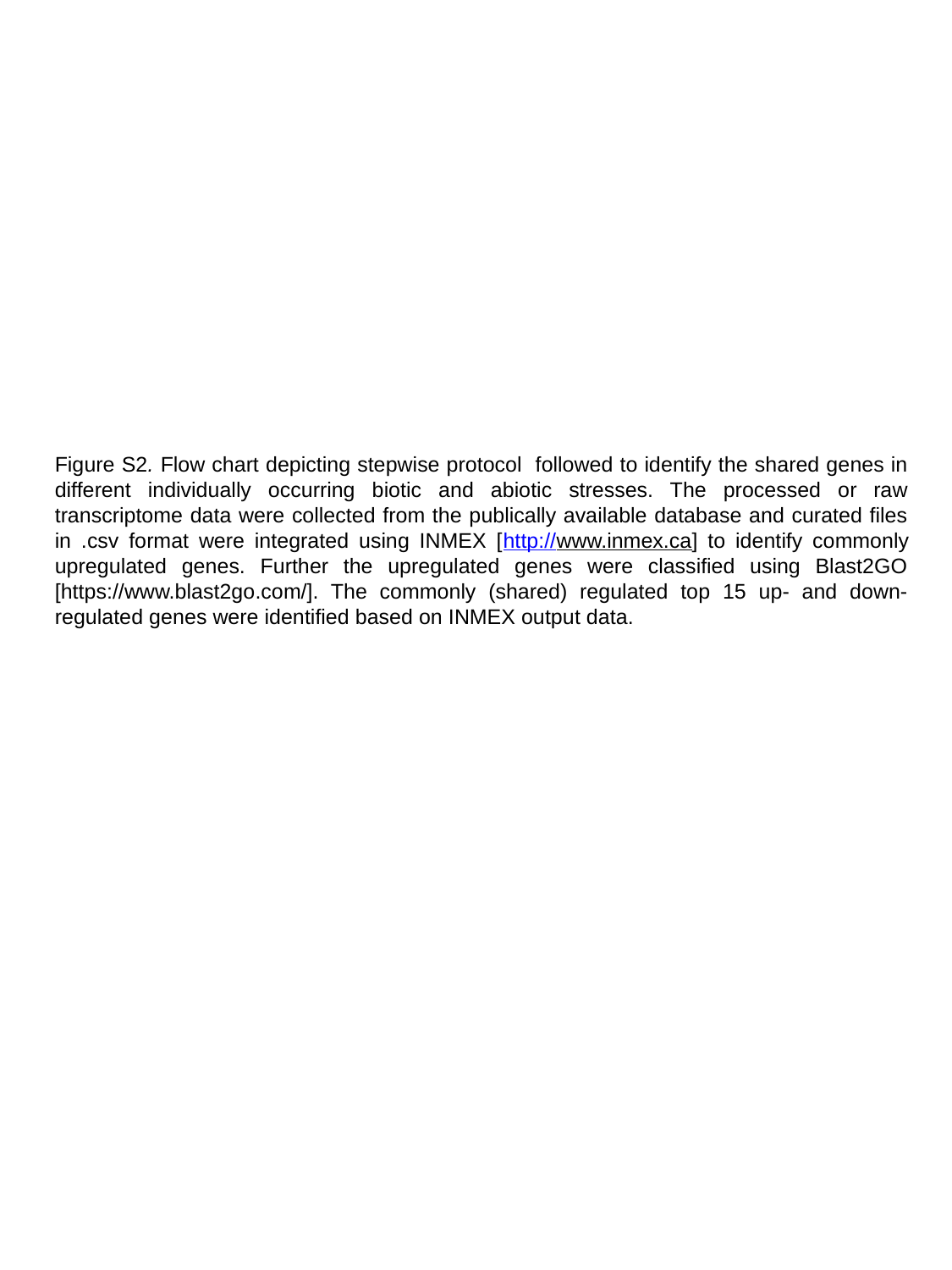

Figure S2. Flow chart depicting stepwise protocol followed to identify the shared genes in different individually occurring biotic and abiotic stresses. The processed or raw transcriptome data were collected from the publically available database and curated files in .csv format were integrated using INMEX [http://www.inmex.ca] to identify commonly upregulated genes. Further the upregulated genes were classified using Blast2GO [https://www.blast2go.com/]. The commonly (shared) regulated top 15 up- and down-regulated genes were identified based on INMEX output data.
