# Supplementary material for: Transcriptome Analysis of Sunflower Genotypes with Contrasting Oxidative Stress Tolerance Reveals Individual- and Combined- Biotic and Abiotic Stress Tolerance Mechanisms
Source: PLoS One. 2016 Jun 17;11(6):e0157522. doi: 10.1371/journal.pone.0157522 (PMC4912118; doi:10.1371/journal.pone.0157522)
Supplement: S3 Fig — (PPT) [file pone.0157522.s003.ppt]

## Slide 1
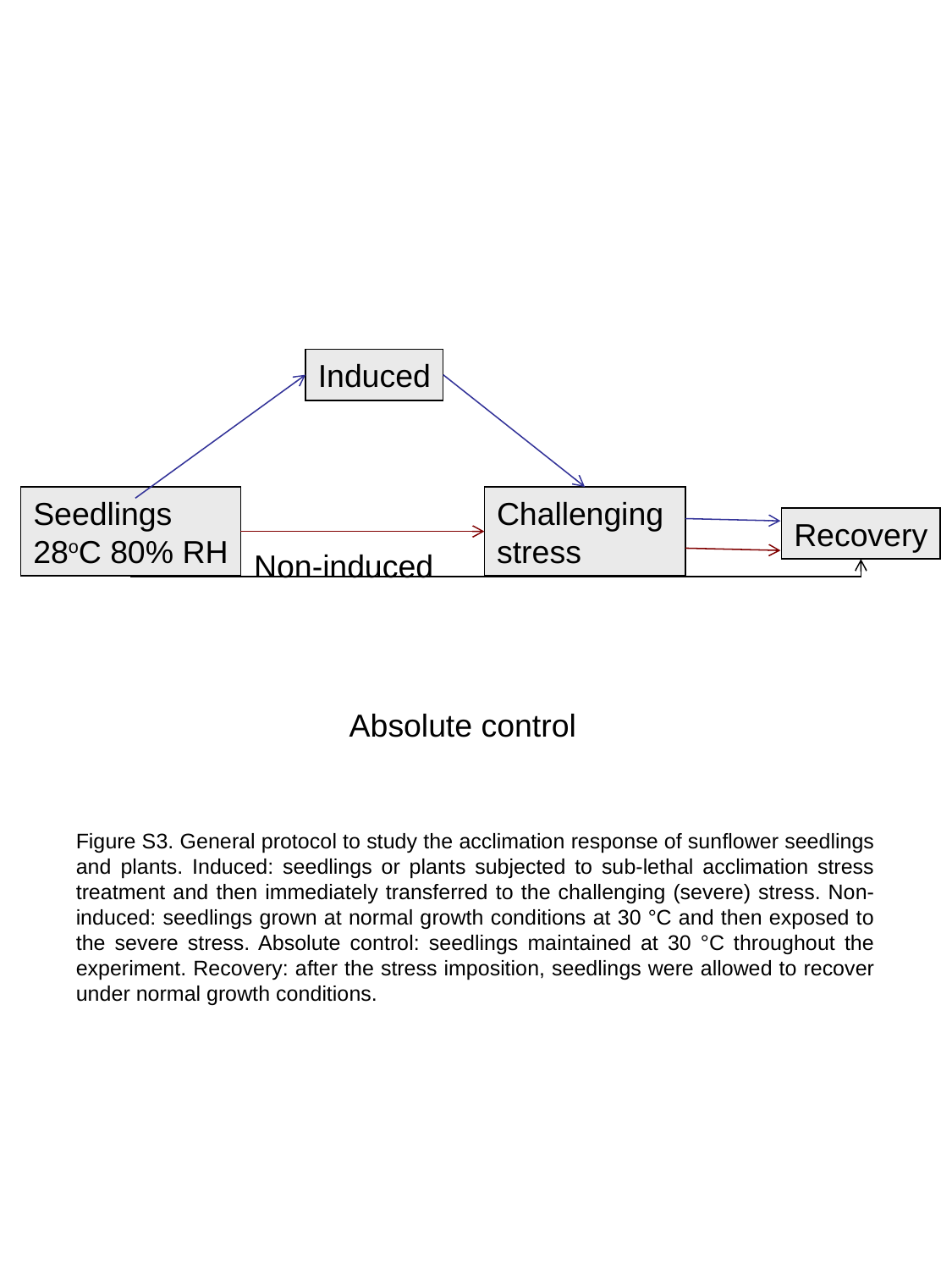

Induced
Seedlings
28oC 80% RH
Challenging stress
Recovery
Non-induced
Absolute control
Figure S3. General protocol to study the acclimation response of sunflower seedlings and plants. Induced: seedlings or plants subjected to sub-lethal acclimation stress treatment and then immediately transferred to the challenging (severe) stress. Non-induced: seedlings grown at normal growth conditions at 30 °C and then exposed to the severe stress. Absolute control: seedlings maintained at 30 °C throughout the experiment. Recovery: after the stress imposition, seedlings were allowed to recover under normal growth conditions.
