# Supplementary material for: Transcriptome Analysis of Sunflower Genotypes with Contrasting Oxidative Stress Tolerance Reveals Individual- and Combined- Biotic and Abiotic Stress Tolerance Mechanisms
Source: PLoS One. 2016 Jun 17;11(6):e0157522. doi: 10.1371/journal.pone.0157522 (PMC4912118; doi:10.1371/journal.pone.0157522)
Supplement: S4 Fig — (PPTX) [file pone.0157522.s004.pptx]

## Slide 1
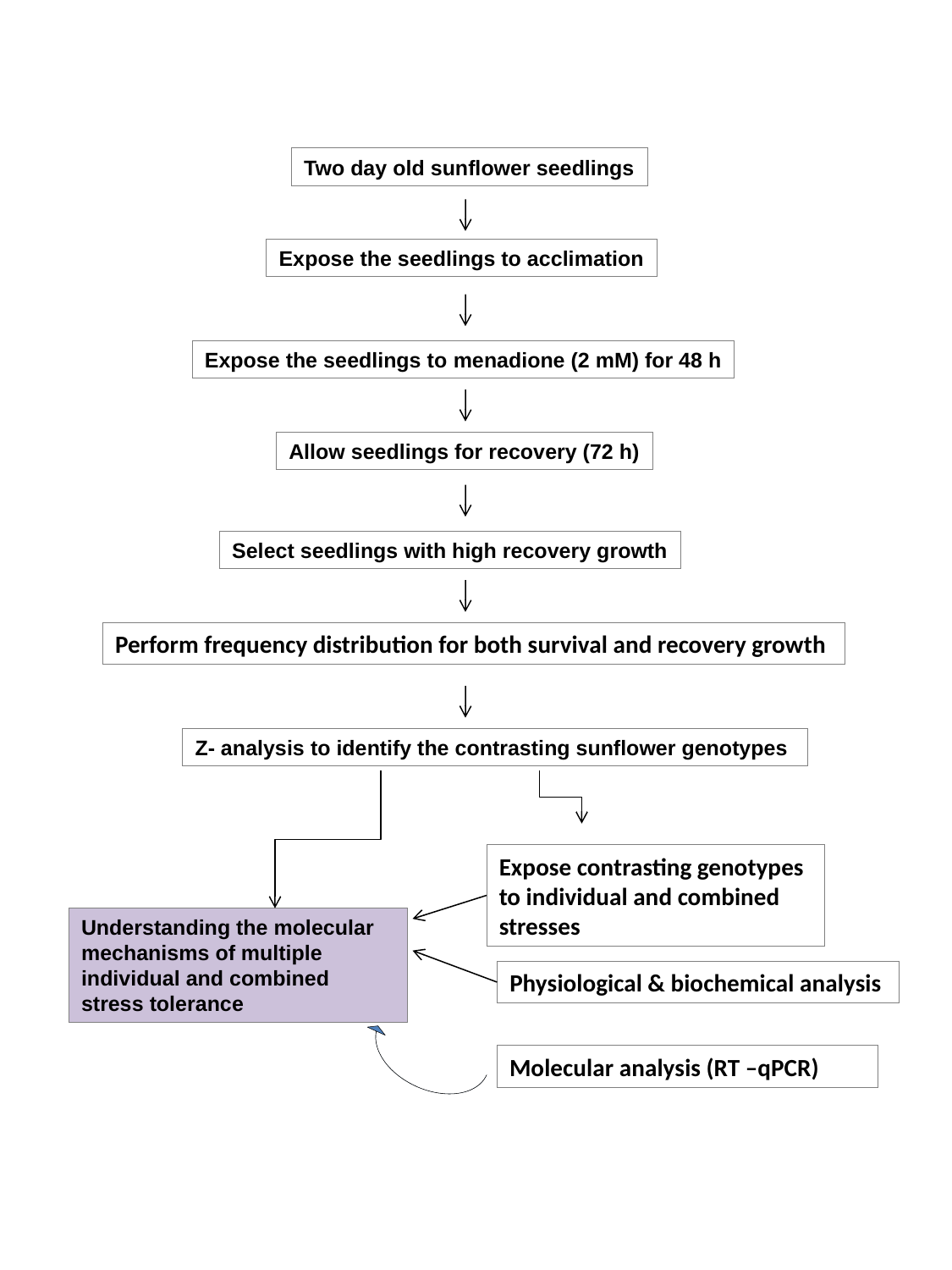

Two day old sunflower seedlings
Expose the seedlings to acclimation
Expose the seedlings to menadione (2 mM) for 48 h
Allow seedlings for recovery (72 h)
Select seedlings with high recovery growth
Perform frequency distribution for both survival and recovery growth
Z- analysis to identify the contrasting sunflower genotypes
Expose contrasting genotypes to individual and combined stresses
Understanding the molecular mechanisms of multiple individual and combined stress tolerance
Physiological & biochemical analysis
Molecular analysis (RT –qPCR)

## Slide 2
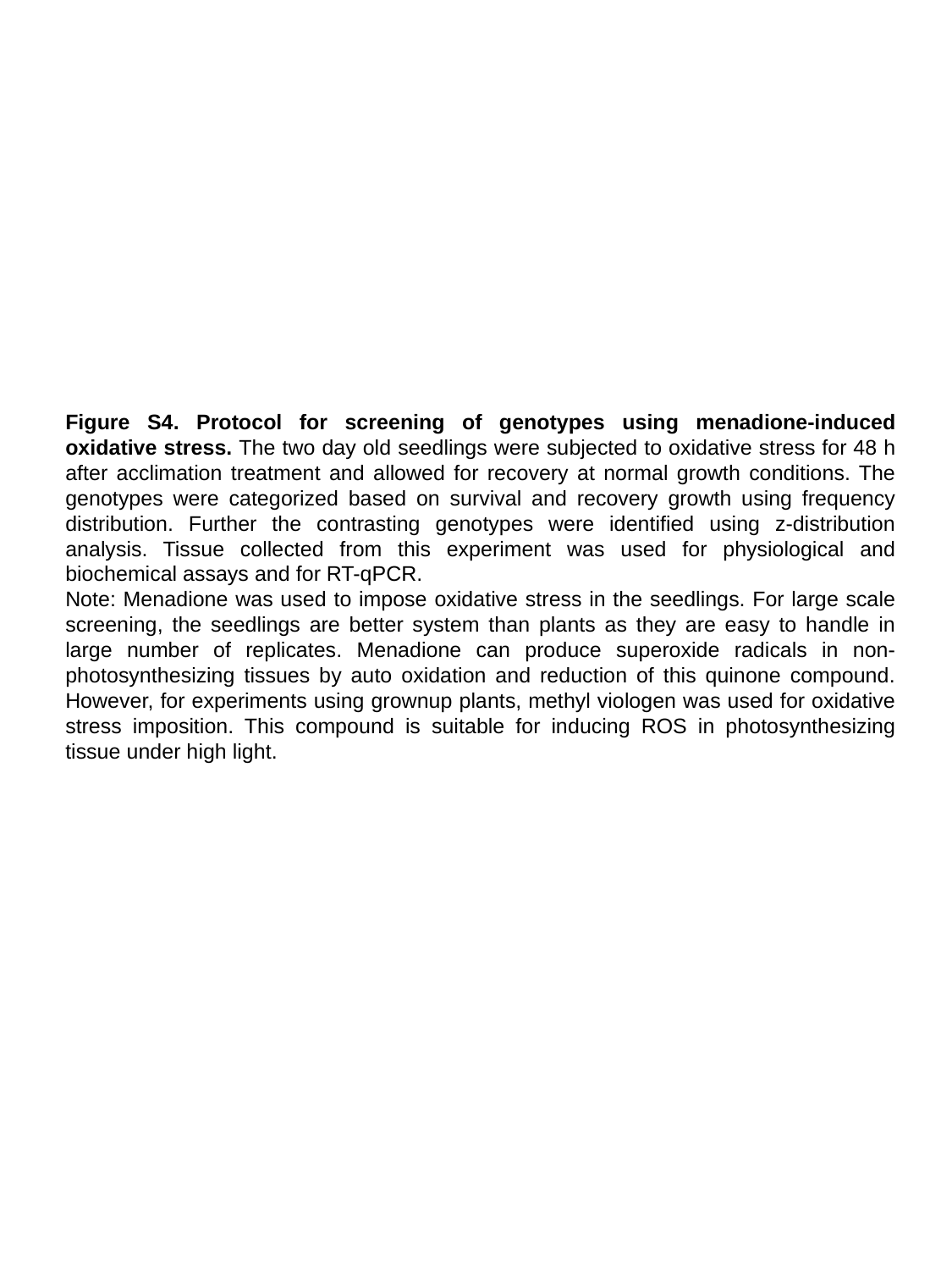

Figure S4. Protocol for screening of genotypes using menadione-induced oxidative stress. The two day old seedlings were subjected to oxidative stress for 48 h after acclimation treatment and allowed for recovery at normal growth conditions. The genotypes were categorized based on survival and recovery growth using frequency distribution. Further the contrasting genotypes were identified using z-distribution analysis. Tissue collected from this experiment was used for physiological and biochemical assays and for RT-qPCR.
Note: Menadione was used to impose oxidative stress in the seedlings. For large scale screening, the seedlings are better system than plants as they are easy to handle in large number of replicates. Menadione can produce superoxide radicals in non-photosynthesizing tissues by auto oxidation and reduction of this quinone compound. However, for experiments using grownup plants, methyl viologen was used for oxidative stress imposition. This compound is suitable for inducing ROS in photosynthesizing tissue under high light.
