# Supplementary material for: Transcriptome Analysis of Sunflower Genotypes with Contrasting Oxidative Stress Tolerance Reveals Individual- and Combined- Biotic and Abiotic Stress Tolerance Mechanisms
Source: PLoS One. 2016 Jun 17;11(6):e0157522. doi: 10.1371/journal.pone.0157522 (PMC4912118; doi:10.1371/journal.pone.0157522)
Supplement: S5 Fig — (PPTX) [file pone.0157522.s005.pptx]

## Slide 1
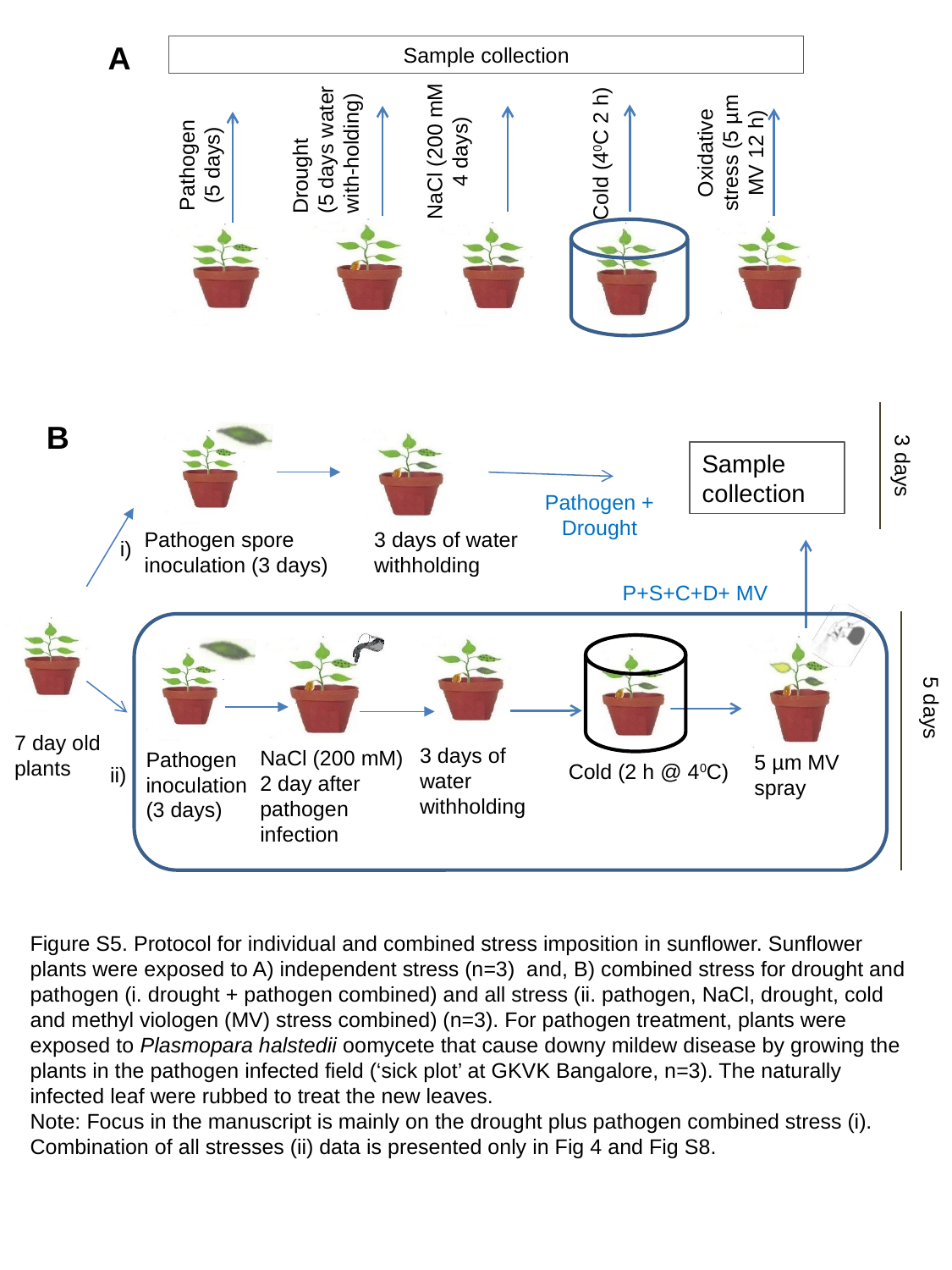

A
Sample collection
Drought
(5 days water with-holding)
Oxidative stress (5 µm MV 12 h)
NaCl (200 mM
4 days)
Cold (40C 2 h)
Pathogen
(5 days)
B
Sample collection
3 days
Pathogen + Drought
3 days of water withholding
Pathogen spore inoculation (3 days)
i)
P+S+C+D+ MV
Cold (2 h @ 40C)
5 days
7 day old plants
3 days of water withholding
NaCl (200 mM)
2 day after pathogen infection
Pathogen inoculation(3 days)
5 µm MV spray
ii)
Figure S5. Protocol for individual and combined stress imposition in sunflower. Sunflower plants were exposed to A) independent stress (n=3) and, B) combined stress for drought and pathogen (i. drought + pathogen combined) and all stress (ii. pathogen, NaCl, drought, cold and methyl viologen (MV) stress combined) (n=3). For pathogen treatment, plants were exposed to Plasmopara halstedii oomycete that cause downy mildew disease by growing the plants in the pathogen infected field (‘sick plot’ at GKVK Bangalore, n=3). The naturally infected leaf were rubbed to treat the new leaves.
Note: Focus in the manuscript is mainly on the drought plus pathogen combined stress (i). Combination of all stresses (ii) data is presented only in Fig 4 and Fig S8.
