# Supplementary material for: Transcriptome Analysis of Sunflower Genotypes with Contrasting Oxidative Stress Tolerance Reveals Individual- and Combined- Biotic and Abiotic Stress Tolerance Mechanisms
Source: PLoS One. 2016 Jun 17;11(6):e0157522. doi: 10.1371/journal.pone.0157522 (PMC4912118; doi:10.1371/journal.pone.0157522)
Supplement: S6 Fig — (PPTX) [file pone.0157522.s006.pptx]

## Slide 1
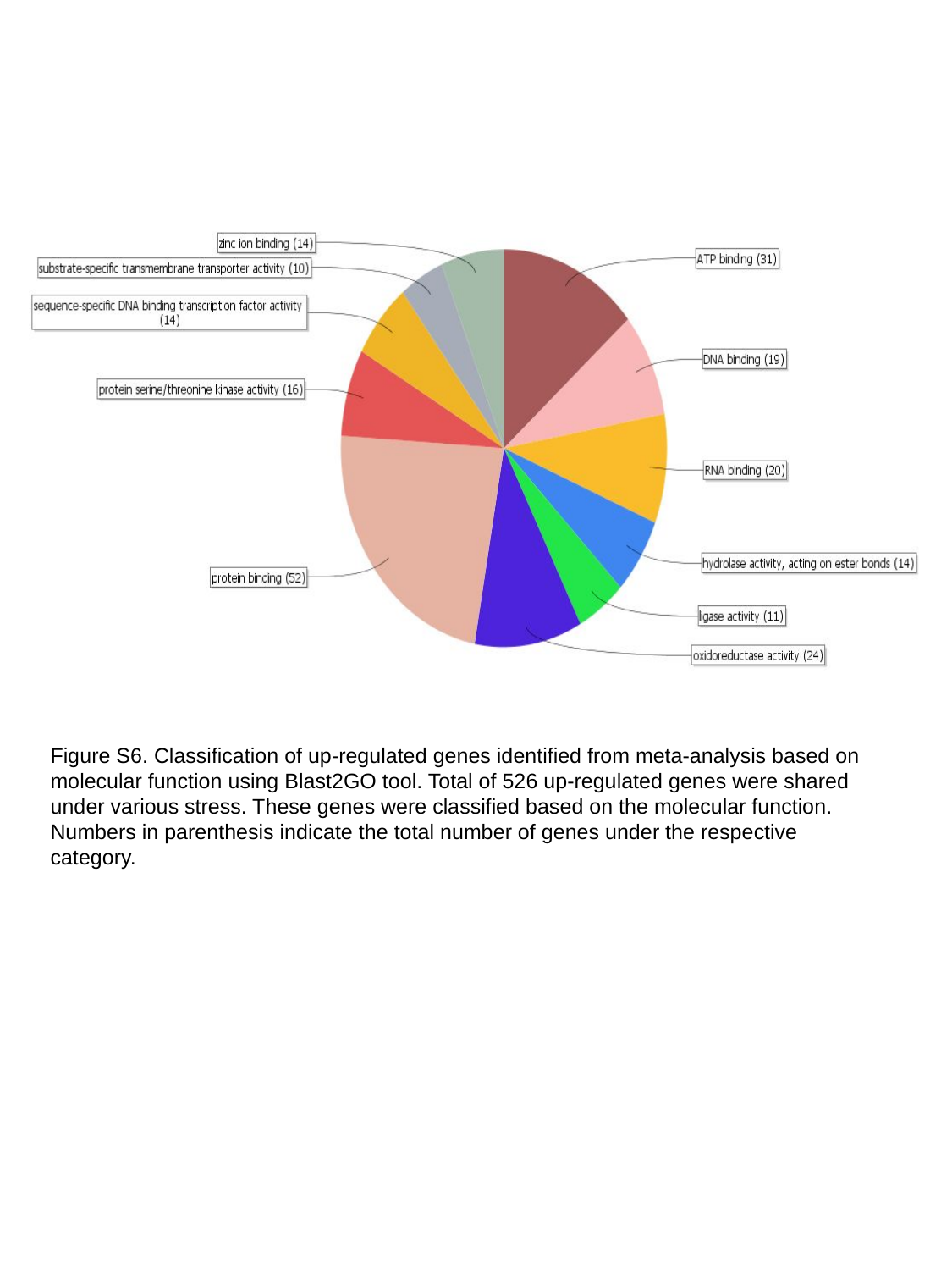

Figure S6. Classification of up-regulated genes identified from meta-analysis based on molecular function using Blast2GO tool. Total of 526 up-regulated genes were shared under various stress. These genes were classified based on the molecular function. Numbers in parenthesis indicate the total number of genes under the respective category.
