# Supplementary material for: Transcriptome Analysis of Sunflower Genotypes with Contrasting Oxidative Stress Tolerance Reveals Individual- and Combined- Biotic and Abiotic Stress Tolerance Mechanisms
Source: PLoS One. 2016 Jun 17;11(6):e0157522. doi: 10.1371/journal.pone.0157522 (PMC4912118; doi:10.1371/journal.pone.0157522)
Supplement: S7 Fig — (PDF) [file pone.0157522.s007.pdf]

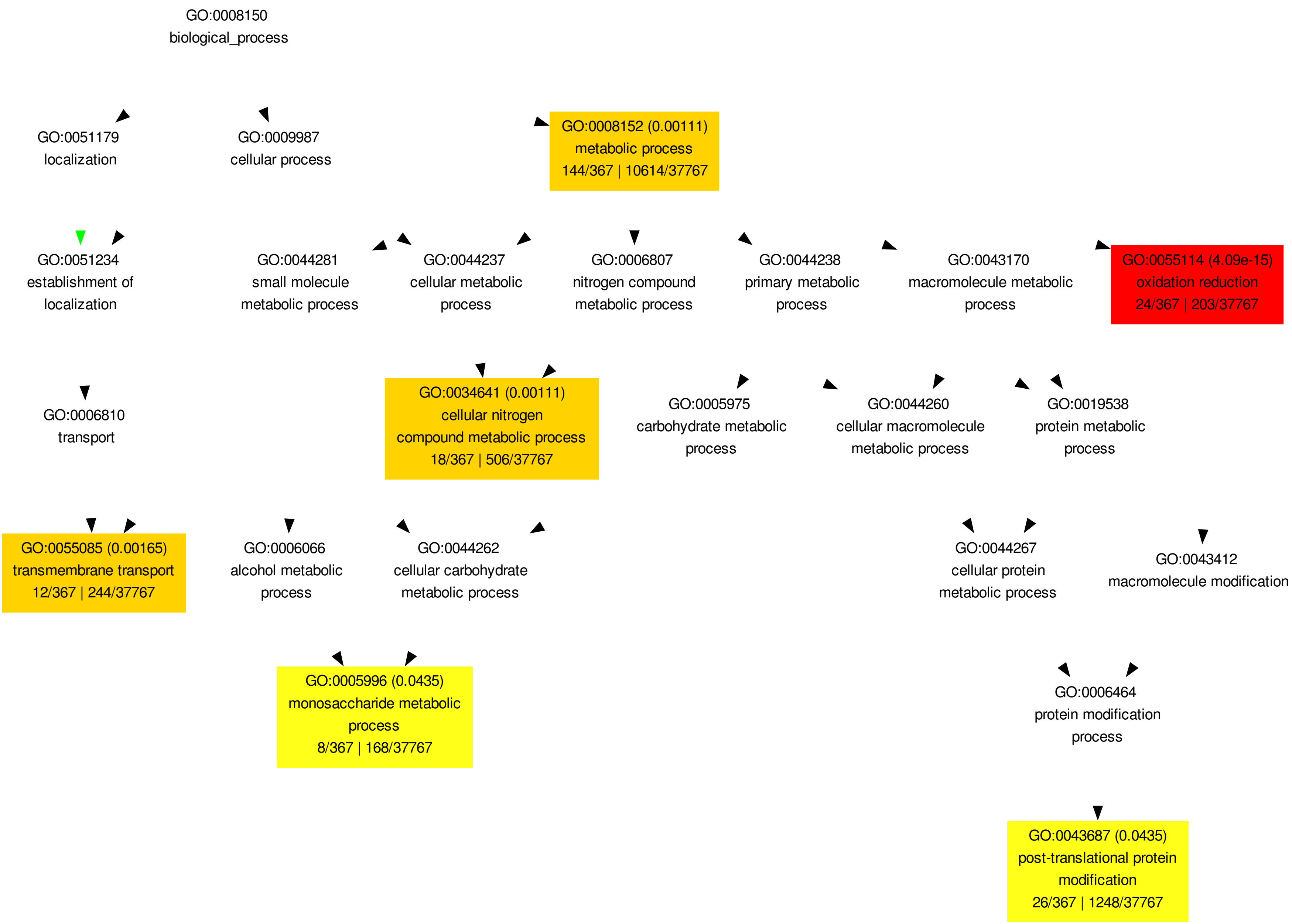

GO:0008150
biological_process
GO:0051179
localization
GO:0009987
cellular process
GO:0008152 (0.00111)
metabolic process
144/367 | 10614/37767
GO:0051234
establishment of
localization
GO:0044281
small molecule
metabolic process
GO:0044237
cellular metabolic
process
GO:0006807
nitrogen compound
metabolic process
GO:0044238
primary metabolic
process
GO:0043170
macromolecule metabolic
process
GO:0055114 (4.09e-15)
oxidation reduction
24/367 | 203/37767
GO:0006810
transport
GO:0034641 (0.00111)
cellular nitrogen
compound metabolic process
18/367 | 506/37767
GO:0005975
carbohydrate metabolic
process
GO:0044260
cellular macromolecule
metabolic process
GO:0019538
protein metabolic
process
GO:0055085 (0.00165)
transmembrane transport
12/367 | 244/37767
GO:0006066
alcohol metabolic
process
GO:0044262
cellular carbohydrate
metabolic process
GO:0044267
cellular protein
metabolic process
GO:0043412
macromolecule modification
GO:0005996 (0.0435)
monosaccharide metabolic
process
8/367 | 168/37767
GO:0006464
protein modification
process
GO:0043687 (0.0435)
post-translational protein
modification
26/367 | 1248/37767

Figure S7. Mapping of upregulated genes based on biological functions. The sunflower GO IDs were used to map using Agrigo software analysis. The colour code of rectangle boxes indicate significance levels yellow=1 and Red=9, Red arrow lines- Positive regulate, green arrow lines= negative regulate, purple line= regulate, yellow line= part of, black dotted lines one or two significant node.
