# Supplementary material for: Transcriptome Analysis of Sunflower Genotypes with Contrasting Oxidative Stress Tolerance Reveals Individual- and Combined- Biotic and Abiotic Stress Tolerance Mechanisms
Source: PLoS One. 2016 Jun 17;11(6):e0157522. doi: 10.1371/journal.pone.0157522 (PMC4912118; doi:10.1371/journal.pone.0157522)
Supplement: S8 Fig — (PDF) [file pone.0157522.s008.pdf]

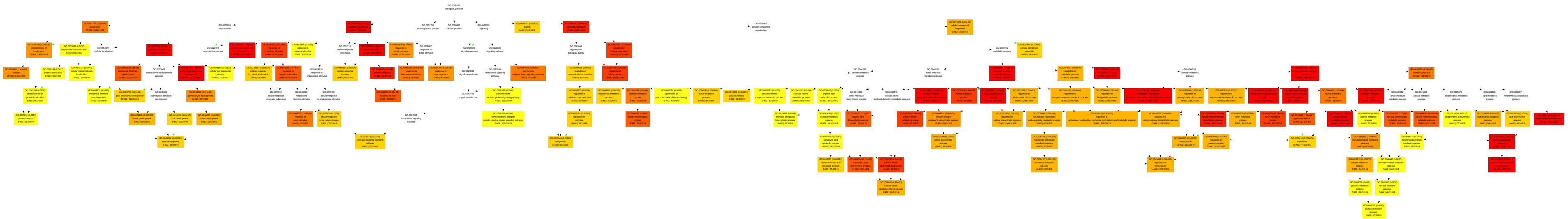

Figure S8. Mapping of downregulated genes based on biological functions. The sunflower GO IDs were used to map using Agrigo software analysis. The colour code of rectangle boxes indicate significance levels yellow=1 and Red=9, Red arrow lines- Positive regulate, green arrow lines= negative regulate, purple line= regulate, yellow line= part of, black dotted lines one or two significant node.
