# Supplementary material for: Transcriptome Analysis of Sunflower Genotypes with Contrasting Oxidative Stress Tolerance Reveals Individual- and Combined- Biotic and Abiotic Stress Tolerance Mechanisms
Source: PLoS One. 2016 Jun 17;11(6):e0157522. doi: 10.1371/journal.pone.0157522 (PMC4912118; doi:10.1371/journal.pone.0157522)
Supplement: S9 Fig — (PPTX) [file pone.0157522.s009.pptx]

## Slide 1
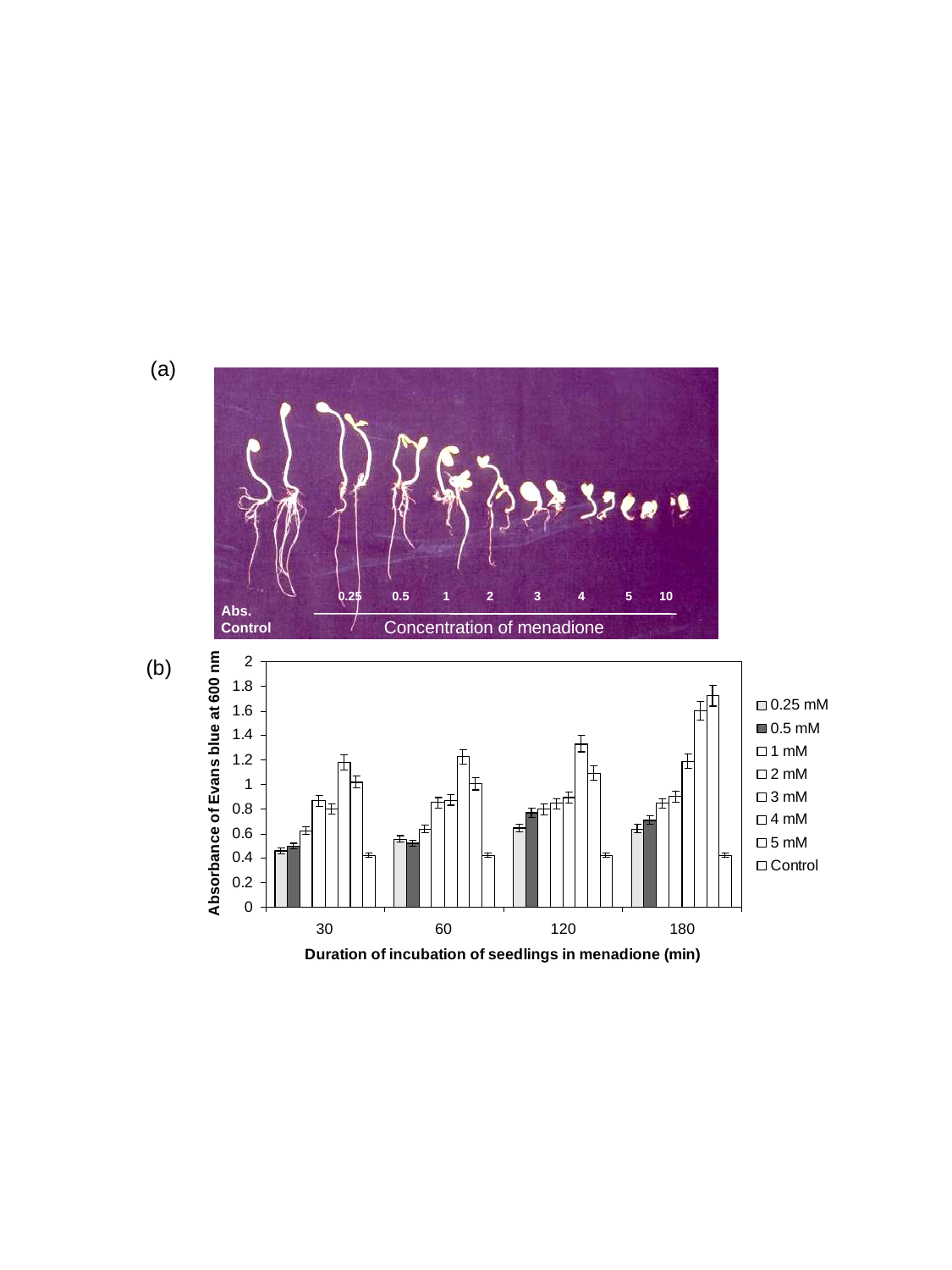

(a)
0.25 0.5 1 2 3 4 5 10
Abs. Control
Concentration of menadione (mM)
(b)

## Slide 2
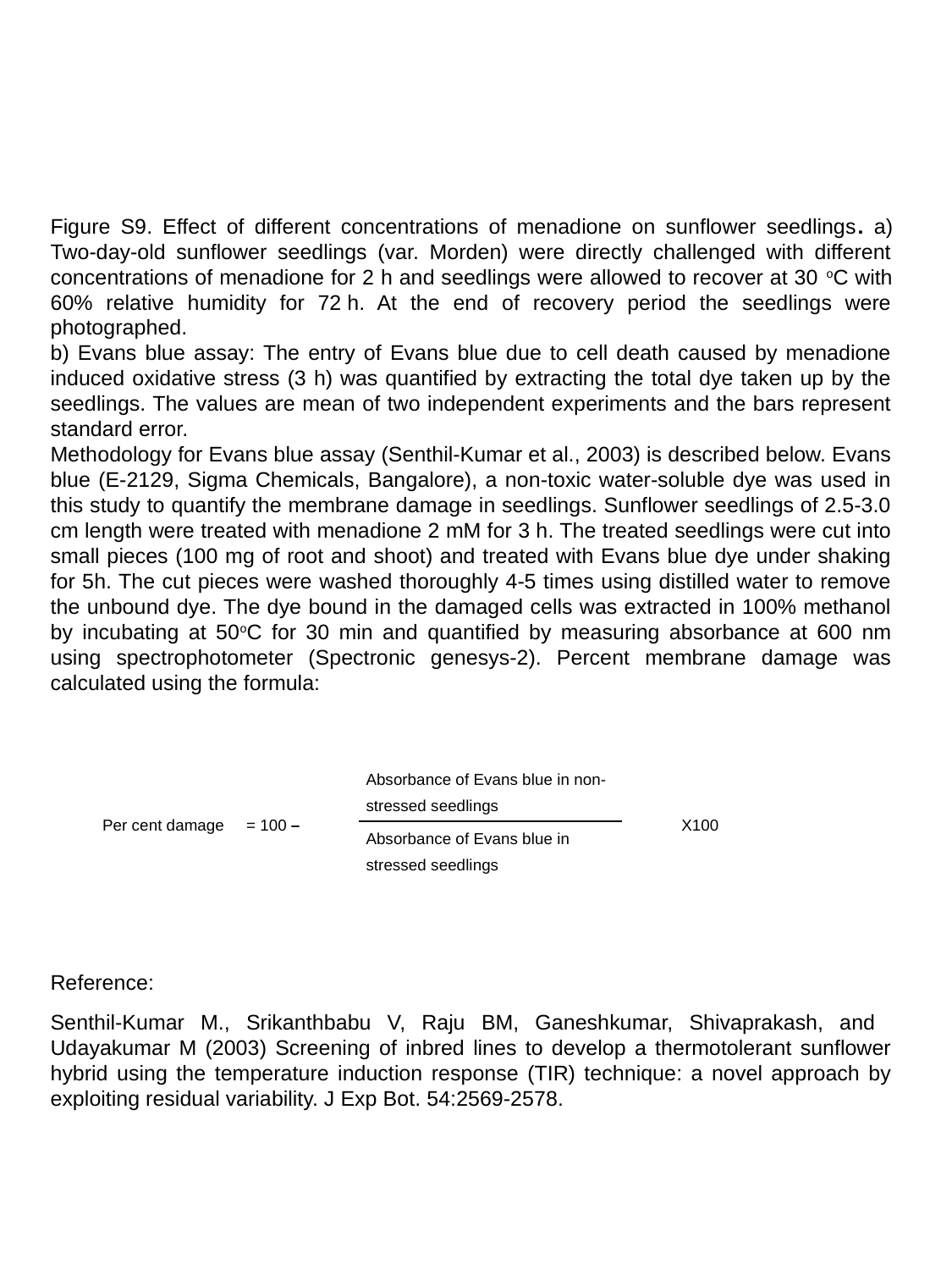

Figure S9. Effect of different concentrations of menadione on sunflower seedlings. a) Two-day-old sunflower seedlings (var. Morden) were directly challenged with different concentrations of menadione for 2 h and seedlings were allowed to recover at 30 oC with 60% relative humidity for 72 h. At the end of recovery period the seedlings were photographed.
b) Evans blue assay: The entry of Evans blue due to cell death caused by menadione induced oxidative stress (3 h) was quantified by extracting the total dye taken up by the seedlings. The values are mean of two independent experiments and the bars represent standard error.
Methodology for Evans blue assay (Senthil-Kumar et al., 2003) is described below. Evans blue (E-2129, Sigma Chemicals, Bangalore), a non-toxic water-soluble dye was used in this study to quantify the membrane damage in seedlings. Sunflower seedlings of 2.5-3.0 cm length were treated with menadione 2 mM for 3 h. The treated seedlings were cut into small pieces (100 mg of root and shoot) and treated with Evans blue dye under shaking for 5h. The cut pieces were washed thoroughly 4-5 times using distilled water to remove the unbound dye. The dye bound in the damaged cells was extracted in 100% methanol by incubating at 50oC for 30 min and quantified by measuring absorbance at 600 nm using spectrophotometer (Spectronic genesys-2). Percent membrane damage was calculated using the formula:
| Per cent damage = 100 – | Absorbance of Evans blue in non-stressed seedlings | | X100 |
| --- | --- | --- | --- |
| | Absorbance of Evans blue in stressed seedlings | | |
Reference:
Senthil-Kumar M., Srikanthbabu V, Raju BM, Ganeshkumar, Shivaprakash, and Udayakumar M (2003) Screening of inbred lines to develop a thermotolerant sunflower hybrid using the temperature induction response (TIR) technique: a novel approach by exploiting residual variability. J Exp Bot. 54:2569-2578.
