# Supplementary material for: Transcriptome Analysis of Sunflower Genotypes with Contrasting Oxidative Stress Tolerance Reveals Individual- and Combined- Biotic and Abiotic Stress Tolerance Mechanisms
Source: PLoS One. 2016 Jun 17;11(6):e0157522. doi: 10.1371/journal.pone.0157522 (PMC4912118; doi:10.1371/journal.pone.0157522)
Supplement: S10 Fig — (PPTX) [file pone.0157522.s010.pptx]

## Slide 1
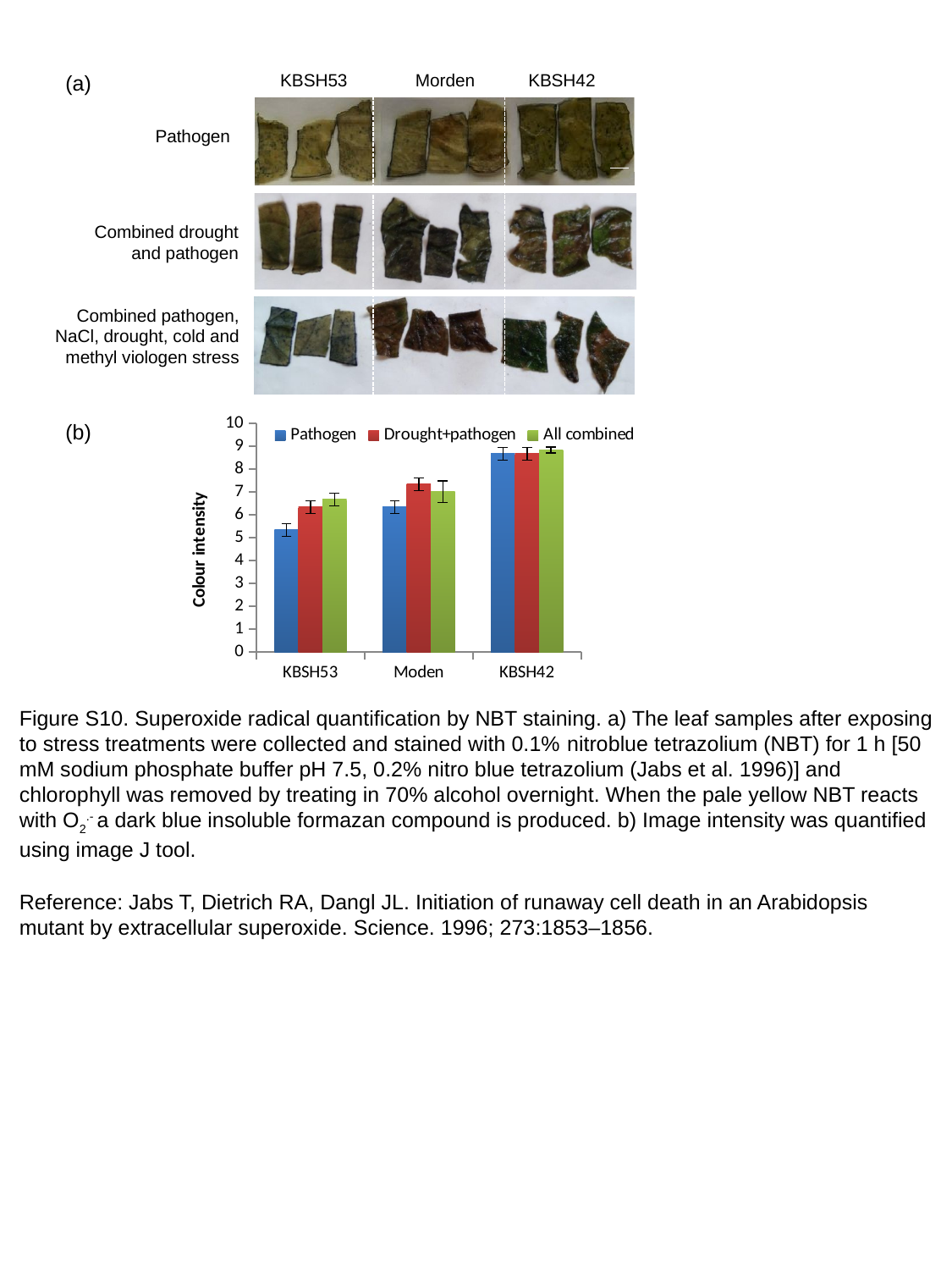

KBSH53 Morden KBSH42
(a)
Pathogen
Combined drought and pathogen
Combined pathogen, NaCl, drought, cold and methyl viologen stress
(b)
### Chart
| Category | Pathogen | Drought+pathogen | All combined |
|---|---|---|---|
| KBSH53 | 5.333333333333332 | 6.333333333333332 | 6.666666666666667 |
| Moden | 6.333333333333332 | 7.333333333333332 | 7.0 |
| KBSH42 | 8.666666666666664 | 8.666666666666664 | 8.833333333333332 |Figure S10. Superoxide radical quantification by NBT staining. a) The leaf samples after exposing to stress treatments were collected and stained with 0.1% nitroblue tetrazolium (NBT) for 1 h [50 mM sodium phosphate buffer pH 7.5, 0.2% nitro blue tetrazolium (Jabs et al. 1996)] and chlorophyll was removed by treating in 70% alcohol overnight. When the pale yellow NBT reacts with O2.- a dark blue insoluble formazan compound is produced. b) Image intensity was quantified using image J tool.
Reference: Jabs T, Dietrich RA, Dangl JL. Initiation of runaway cell death in an Arabidopsis mutant by extracellular superoxide. Science. 1996; 273:1853–1856.
