# Supplementary material for: Transcriptome Analysis of Sunflower Genotypes with Contrasting Oxidative Stress Tolerance Reveals Individual- and Combined- Biotic and Abiotic Stress Tolerance Mechanisms
Source: PLoS One. 2016 Jun 17;11(6):e0157522. doi: 10.1371/journal.pone.0157522 (PMC4912118; doi:10.1371/journal.pone.0157522)
Supplement: S1 Table — (DOCX) [file pone.0157522.s011.docx]

Table S1: Genetic background, agronomic characteristics testing locations of sunflower varieties and hybrids used for screening in this study.

| **Sl. No.** | **Entry code No.** | **Variety/ hybrid name** | **Plant height (cm)** | **Head diameter (cm)** | **Days for 50% flowering** | **Seed yield (Kg/ha)** | **Oil content (%)** | **Genetic nature** | **Response to menadione stress** | **Location & possible stress exposure during growth season**** |
| --- | --- | --- | --- | --- | --- | --- | --- | --- | --- | --- |
| 1 | IAVT 01 | LSF-300 | 136 | 15.4 | 59 | 773 | 32.7 | Pre-released variety | Extremely Sensitive | Bangalore, Karnataka; various fungal pathogens, including those causing mildews and virus diseases; Raichur, Karnataka, Drought and temperature extreme |
| 2 | IAVT 02 | SS-2038 | 160 | 14.9 | 57 | 1154 | 31.3 | Pre-released variety | Extremely Sensitive |  |
| 3 | IAVT 04 | RSFV-901-1 | 154 | 12.8 | 62 | 598 | 28.9 | **Variety** | Extremely Sensitive |  |
| 4 | IAVT 05 | GAUSUF-12 | 180 | 15.4 | 63 | 760 | 30.6 | Pre-released variety | Moderate resistance |  |
| 5 | IAVT 06 | DRSF-118 | 167 | 15.9 | 59 | 669 | 28.3 | **Variety** | Sensitive |  |
| 6 | IAVT 07 | DRSF-119 | 183 | 17 | 56 | 1113 | 40.5 | Pre-released variety | Sensitive |  |
| 7 | IAVT 08 | TNAUSUF-7 | 179 | 15.5 | 58 | 822 | 26.8 | **Variety** | Extremely Sensitive |  |
| 8 | IAVT 09 | GAUSUF-15 | 156 | 18.2 | 57 | 801 | 29.4 | **Variety** | Extremely Sensitive |  |
| 9 | IAVT 10 | DRSF-108 | 156 | 15.1 | 61 | 783 | 30.4 | **Variety** | Extremely Sensitive |  |
| 10 | IAVT 11 | Morden | 138 | 14.6 | 53 | 1047 | 24.3 | **Population/ Variety** | Extremely Sensitive |  |
| 11 | AHT 02 | JKSFH-238 | 170 | 15.6 | 55 | 1434 | 38.5 | Pre-released hybrid | Resistant | Akola, Maharashtra; drought |
| 12 | AHT 03 | KBSH-44 | 150 | 16.4 | 65 | 778 | 20.1 | **Hybrid** | Resistant |  |
| 13 | AHT 04 | PAC-1091 | 154 | 16.1 | 63 | 757 | 27.4 | **Hybrid** | Resistant |  |
| 14 | AHT 05 | K-642 | 161 | 15.4 | 65 | 1110 | 37.2 | Pre-released hybrid | Moderate |  |
| 15 | IHT690 | DRSH-105 | 178 | 15.3 | 59 | 1435 | 40.8 | Hybrid under initial testing | Resistant | Coimbatore, Tamil Nadu; Nandyal, Andhra Pradesh, Drought and temperature variations; Nimpith, West Bengal, Salinity |
| 16 | IHT691 | BISCO-209 | 175 | 14.8 | 66 | 1421 | 39.4 | Hybrid under initial testing | Resistant |  |
| 17 | IHT692 | Sunbred 19012 | 154 | 15.1 | 60 | 1806 | 41.0 | Hybrid under initial testing | Resistant |  |
| 18 | IHT693 | DRSH-405 | 164 | 15.6 | 55 | 1284 | 37.5 | Hybrid under initial testing | Resistant |  |
| 19 | IHT695 | 64S-99 | 170 | 14.5 | 65 | 1667 | 42.0 | **Hybrid** | Resistant |  |
| 20 | IHT696 | KBSH-55 | 182 | 14.7 | 55 | 1099 | 37.9 | **Hybrid** | Resistant |  |
| 21 | IHT697 | KBSH-437 | 128 | 14.2 | 60 | 969 | 20.9 | Hybrid under initial testing | Resistant |  |
| 22 | IHT698 | CSFH-5195 | 156 | 14.7 | 60 | 788 | 27.8 | Hybrid under initial testing | Resistant |  |
| 23 | IHT699 | LSFH-05-36 | 173 | 14.5 | 56 | 1218 | 39.2 | Hybrid under initial testing | Resistant |  |
| 24 | IHT700 | KBSH-56 | 168 | 15.2 | 54 | 790 | 33.9 | **Hybrid** | Resistant |  |
| 25 | IHT701 | PKVSH-58 | 117 | 13.5 | 53 | 1439 | 37.5 | Hybrid under initial test164ing | Sensitive |  |
| 26 | IHT702 | KBSH-1 | 175 | 14.6 | 57 | 1468 | 42.0 | **Hybrid** | Resistant |  |
| 27 | IHT703 | PAC-1091 | 164 | 15.2 | 62 | 1332 | 31.0 | **Hybrid** | Resistant |  |
| 28 | IHT704 | KBSH-44 | 156 | 14.9 | 65 | 903 | 22.2 | **Hybrid** | Moderate Resistant |  |
| 29 | IHT705 | Sunbred 00997 | 162 | 16.1 | 65 | 1826 | 42.4 | Hybrid under initial testing | Resistant |  |
| 30 | IHT706 | PAC-361 | 178 | 17.4 | 68 | 1236 | 39.5 | Hybrid under initial testing | Resistant |  |
| 31 | IHT707 | JK Chitra | 160 | 16.1 | 66 | 1186 | 34.2 | **Hybrid** | Sensitive |  |
| 32 | IHT708 | BISCO-210 | 150 | 14.9 | 55 | 1527 | 37.8 | Hybrid under initial testing | Extremely Sensitive |  |
| 33 | IHT709 | PAC-1091 | 150 | 16.7 | 64 | 873 | 25.3 | **Hybrid** | Resistant |  |
| 34 | IHT711 | KBSH-1 | 163 | 15.6 | 58 | 1146 | 38.0 | **Hybrid** | Sensitive |  |
| 35 | IHT712 | KSFH-446 | 154 | 14.8 | 51 | 1199 | 34.6 | Hybrid under initial testing | Sensitive |  |
| 36 | IHT714 | NSH-391 | 144 | 16.0 | 61 | 530 | 31.8 | Hybrid under initial testing | Sensitive |  |
| 37 | IHT715 | MDSFH-404 | 156 | 15.8 | 54 | 1312 | 37.8 | Hybrid under initial testing | Extremely sensitive |  |
| 38 | IHT716 | BSFH-4 | 162 | 15.7 | 60 | 1354 | 35.6 | Hybrid under initial testing | Resistant |  |
| 39 | IHT717 | Hybrid Sunflower 3389 | 164 | 15.4 | 55 | 1382 | 36.2 | Hybrid under initial testing | Resistant |  |
| 40 | IHT718 | ARSH-496 | 158 | 15.2 | 64 | 561 | 35.0 | Hybrid under initial testing | Sensitive |  |
| 41 | IHT719 | KHS-2010 | 133 | 16.0 | 62 | 848 | 35.0 | Hybrid under initial testing | Extremely sensitive |  |
| 42 | IHT720 | KHS-2030 | 150 | 15.9 | 61 | 1335 | 37.4 | Hybrid under initial testing | Extremely sensitive |  |
| 43 | S-3262 | - | - | - | - | - | - | Pre-released variety | Resistant | Dholi, Bihar; Temperature related stress |
| 44 | S-2077 | - | - | - | - | - | - | Pre-released variety | Resistant |  |
| 45 | S-Atul | - | - | - | - | - | - | Pre-released variety | Sensitive |  |
| 46 | SSFH1005 | - | - | - | - | - | - | Pre-released hybrid | Resistant |  |
| 47 | 64 S 99 | - | - | - | - | - | - | Pre-released hybrid | Resistant |  |
| 48 | SH 416 | - | - | - | - | - | - | Pre-released hybrid | Moderate resistance | Hisar, Haryana; Temperature related stress |
| 49 | 64 A 57 | - | - | - | - | - | - | Pre-released hybrid | Moderate resistance |  |
| 50 | Sunflower Hybrid (SH) 6068 | - | - | - | - | - | - | Pre-released hybrid | Moderate resistance |  |
| 51* | Modern^#^ | - | 132 | 14.0 | 52 | 950 | 24.5 | **Population/ variety** | Resistant | Raichur, Karnataka, Drought and temperature extreme |
| 52* | KBSH-53^#^ | - | 187 | 14.0 | 69 | 1733 | 39.4 | **Hybrid** | Resistant |  |
| 53* | KBSH-42^#^ | - | 185 | 14.0 | 63 | 1256 | 33.2 | **Hybrid** | Sensitive |  |
| 54* | KBSH-41^#^ | - | 197 | 14.6 | 61 | 1309 | 35.9 | **Hybrid** | Sensitive |  |
| 55* | KBSH-44^#^ | - | 179 | 14.2 | 65 | 956 | 30.7 | **Hybrid** | Moderate resistant |  |

*seeds were obtained from ‘All India Co-ordinated Research Project on Sunflower (AICRP-SF)’ at AICRP Sunflower Unit, Regional Research Station, University of Agricultural Sciences (UAS), GKVK Bangalore India.

All other seeds were obtained from National Seeds Project, UAS, GKVK, Bangalore India.

**information derived based on various public resources

IAVT- Initial and advanced varietal trial

IHT- Initial hybrid trial

AHT- Advanced hybrid trial

KBSH- Karnataka Bangalore Sunflower hybrid

SH - Sunflower Hybrid

^#^**additional details**

| Modern | Short duration dwarf; 75 days maturity | Cultivated variety |
| --- | --- | --- |
| KBSH-53 | Short duration (90-95 days) dwarf , 1700-2700 kg/ha seed, 42--44% oil content, 700-1000 kg/ha oil yield | CMS 335A × RHA95C-1 Resistance to powdery mildew and others |
| KBSH-42 | Short duration (90-95 days) dwarf , 1600-2350 kg/ha seed, 39-41% oil content, 650-900 kg/ha oil yield | CMS 851A × RHA 95 C-1  Susceptible to powdery mildew |
| KBSH-41 | Short duration (90-92 days) dwarf, 1700-2500 kg/ha seed yield, 40-42% oil content, 650-900 kg/ha oil yield | CMS234A × RHA95C-1  Susceptible to powdery mildew |
| KBSH-44 | Short duration (95-100 days) dwarf, 1500-2800 kg/ha seed, 36-38% oil content, 650-1000 kg/ha oil yield | CMS CMS17A× RHA95C-1 Susceptible to powdery mildew |
