# Supplementary material for: Transcriptome Analysis of Sunflower Genotypes with Contrasting Oxidative Stress Tolerance Reveals Individual- and Combined- Biotic and Abiotic Stress Tolerance Mechanisms
Source: PLoS One. 2016 Jun 17;11(6):e0157522. doi: 10.1371/journal.pone.0157522 (PMC4912118; doi:10.1371/journal.pone.0157522)
Supplement: S2 Table — (DOCX) [file pone.0157522.s012.docx]

Table S2. Details of sunflower tissue, stress treatment and microarray source used in the transcriptomic data downloaded from public sources.

| **Sl. No** | **Name of array** | **Type of stress** | **Age of plant and plant part used** | **Stress treatment and duration** | **Reference** |
| --- | --- | --- | --- | --- | --- |
| 1 | E-GEOD-45586 | Cold | Effect of cold stress 6 leaves well-developed stage, 30-35 days old, leaf sample | 1. Cold acclimation (+4°C during 2 days) followed by 2 nights at -3°C (frost treatment)  2. 23°C day/18°C night, 63% humidity, 14 h day photoperiod | <https://www.ebi.ac.uk/arrayexpress/experiments/E-GEOD-45586/?query=%22Helianthus+annuus%22+> |
| 2 | E-GEOD-44165 | ROS and hormone | Dormant, non-dormant seed, embryo | Dormant and non-dormant sunflower embryo imbibed 24 h on water, on ABA, on methylviologen, a pro-oxidant compound or on ethylene. | <https://www.ebi.ac.uk/arrayexpress/experiments/E-GEOD-44165/?query=%22Helianthus+annuus%22+> |
| 3 | E-GEOD-25717-710 | Pathogen | Effect of downy mildew  6-10 day old cotyledons, first two leaves inoculated | ***Plasmopara halstedii -*** Race-710  Time-3 h  Polygonal spots occur on the above ground parts, especially on leaves. | <https://www.ebi.ac.uk/arrayexpress/experiments/E-GEOD-25717/?query=%22Helianthus+annuus%22+> |
| 4 | E-GEOD-25717-334 | Pathogen | Effect of downy mildew  6-10 day old cotyledons, first two leaves inoculated | ***Plasmopara halstedii -*** Race-334  Time-3 h  Polygonal spots occur on the above ground parts, especially on leaves. | <https://www.ebi.ac.uk/arrayexpress/experiments/E-GEOD-25717/?query=%22Helianthus+annuus%22+> |
| 5 | E-GEOD-22519 | ABA/ drought | Identification of ABA regulated genes 12 day old leaves | ABA-10 μM for 6 h  Growth conditions were 14 h light at 23°C and 10 h night at 20°C under fluorescent bulbs. Plants were grown in 6 hydroponic boxes containing 20 litres of aerated liquid culture medium | <https://www.ebi.ac.uk/arrayexpress/experiments/E-GEOD-22519/?query=%22Helianthus+annuus%22+> |
| 6 | E-GEOD-36304 | Drought | Assessing drought stress in field grown plants 86 day old flowering plant | **Drought stress**- No Water  **Control**- Watered once in 30 days | <https://www.ebi.ac.uk/arrayexpress/experiments/E-GEOD-36304/?query=%22Helianthus+annuus%22+> |
| 7 | E-GEOD-38812 | ABA/drought | Identification  of for QTL of ABA response , 15 day old plantlets | ABA-0.5 μM for 6 h  Growth conditions were 14 h light at 23°C and 10 h night at 18°C under fluorescent bulbs | <https://www.ebi.ac.uk/arrayexpress/experiments/E-GEOD-38812/?query=%22Helianthus+annuus%22+> |
