# Supplementary material for: Transcriptome Analysis of Sunflower Genotypes with Contrasting Oxidative Stress Tolerance Reveals Individual- and Combined- Biotic and Abiotic Stress Tolerance Mechanisms
Source: PLoS One. 2016 Jun 17;11(6):e0157522. doi: 10.1371/journal.pone.0157522 (PMC4912118; doi:10.1371/journal.pone.0157522)
Supplement: S3 Table — (DOCX) [file pone.0157522.s013.docx]

Table S3. List of genes and primer sequences used in the study

| **Sl. No.** | **Gene** | **Primer location** | **Sequence (5′-3′)*** |
| --- | --- | --- | --- |
| 1 | *C2H-ZF* | FP | CTGGGCATCAAGCCCCACAG |
|  |  | RP | TTCACGTCGCTGCTCTCCTG |
| 2 | *Chitinase* | FP | TACTGCGACGAGAACAACACCCAG |
|  |  | RP | GCTCAGGATCACGCTGTGCA |
| 3 | *MYB TF* | FP | GTTCAAGCCCACCGTGAGGA |
|  |  | RP | TTCCTCACGGGGAACAGCTCG |
| 4 | *MI-1-PS* | FP | CGACGACTTCAAGAGCGGCCA |
|  |  | RP | GTACAGGATGGCGTTGCTGTTCAC |
| 5 | *MYC2* | FP | GAGTTCAGGCTGAGCCAGTTCC |
|  |  | RP | TCCACGTGGTTCAGGGGCTC |
| 6 | *CdCIPK2* | FP | AGGCTGAAGCAGTTCAGCGTG |
|  |  | RP | GCCTCCATCAGGATCTGCACGTC |
| 7 | *PR5-1* | FP | ACGTGCCCATGGTGTTCAGG |
|  |  | RP | CCTGGTCTTGAAGAACCTGCTCAG |
| 8 | *PAP1* | FP | GCCAAGTGGAAGATCGTGGTG |
|  |  | RP | TCATGTCGCCCCTCCAGCTC |
| 9 | *LTP3* | FP | AGCTTCAGGACCCACCAGATCGA |
|  |  | RP | CAGCTGGTCTGGGTCAGCCA |
| 10 | *ERF12* | FP | CAAGGCCAAGACCAACTTCC |
|  |  | RP | TCTTCACGGGGATGGGCTCG |
| 11 | *LEA14* | FP | CAAGGGCAAGATCCCCGACC |
|  |  | RP | GGGATGGTGAACTCGCCCAC |
| 12 | *ERD6* | FP | CCCCGTGGAGTACCACAACG |
|  |  | RP | GGTCTCCTTGAACAGCAGCACCA |
| 13 | *Helicase* | FP | AGCGTGAAGTTCCACGCCGA |
|  |  | RP | TCCACGCTGGGGATGTCCAG |
| 14 | *DEAD box helicase* | FP | TTCCTGGGCAGCGTGAAGGA |
|  |  | RP | ATCTGGCCCTCGATGTGGCTG |
| 15 | *Put, Dehydrogenase* | FP | ACAGGAGCATCCTGCTGTTCGA |
|  |  | RP | CAGCTTCTCGTAGCCGCTGTTCTC |
| 16 | *DnaJ* | FP | ACGACCTGTTCGTGGAGCACAC |
|  |  | RP | TCGGGGAACTCCACGGTGAA |
| 17 | *Chaperonin* | FP | ACATCGTGGGCATCCTGGAGAC |
|  |  | RP | TCTTGCCGTCCTCGTCCAG |
| 18 | *BA-P12* | FP | TGCTTCGGCTTCCAGGAGCA |
|  |  | RP | TCCTGGCTCAGGTCGATGGTG |
| 19 | *O-6FAD* | FP | GCCCCATCTACAACGACAGGGA |
|  |  | RP | ACTCGCTGCTGTCGTAGTG |
| 20 | *H2AZ* | FP | AAGGGCAGGTACGCCCAGAG |
|  |  | RP | ACGGGGTTGATGTTGGGCAG |
| 21 | *Mg Chelatase* | FP | GTGCTGTGGATGATCGGCGTGAA |
|  |  | RP | AGGGCGTGCTTCCTCACGAA |
| 22 | *DNA topo 2* | FP | GTGAACCTGCTGCAGCCCAA |
|  |  | RP | ACGTACCAGGTGGGCTCGATGTA |
| 23 | *LHCP* | FP | AGCTGGAGGTGATCCACTGC |
|  |  | RP | CCCATCAGGATCACCTGGGTG |
| 24 | *TiPa* | FP | ATGGAGGTGATCCTGACCTTCAGC |
|  |  | RP | GTCCAGATGCCGCTCACGAA |
| 25 | *Cys-γ syn* | FP | AGGAACCTGCACCACATCCTGG |
|  |  | RP | AAGCCGGTCATCTGCTTCTTGG |
| 26 | *ARP8c* | FP | CGACAGGATCCCCGTGATCGTG |
|  |  | RP | GTTCTCGTCGTAGATGGCGCTC |
| 27 | *RL10* | FP | CAACACCAGCAACACCGGCA |
|  |  | RP | GGTCAGGCTCCTGTAGGCCA |
| 28 | *HxK2* | FP | ACTACCTGGGCGAGATCCTG |
|  |  | RP | AGCCTCCTGATCAGCTTCCTC |
| 29 | *PSI reaction centre* | FP | ATCAAGGCCCAGCAGGTGAG |
|  |  | RP | GTGCCGAACTGCACGGTGTA |
| 30 | *SOD* | FP | TTGGCAATGTCACAGTAGGC |
|  |  | RP | CCCTGCAATCCAATGATACC |
| 31 | *APX* | FP | GGAGTTGTTGCTGTGGAGGT |
|  |  | RP | TGTGCTCTTCCCAGAGTGTG |
| 32 | *CATALASE* | FP | ACACTCAGAGGCACCGTCTT |
|  |  | RP | GCTTTCCGGACAACCTAACA |
| 33 | *HSP* | FP | CGTCGTCTCCATCAAGGTTT |
|  |  | RP | CTGCTTCCCTAACCATACGC |
| 34 | *Actin* | FP | AGCTGCTGGTATTCACGAGACC |
|  |  | RP | TCGATCCTCCGATCCAGACACTG |
| 35 | *Ubiquitin* | FP | \| AGCAAAGGCTCATCTTTGCAGG \| \| --- \| |
|  |  | RP | GATAGTGTCCGAGCTCTCCA |

*primer sequences were used for performing RT-qPCR

FP, forward primer; RP, reverse primer
