# Supplementary material for: Transcriptome Analysis of Sunflower Genotypes with Contrasting Oxidative Stress Tolerance Reveals Individual- and Combined- Biotic and Abiotic Stress Tolerance Mechanisms
Source: PLoS One. 2016 Jun 17;11(6):e0157522. doi: 10.1371/journal.pone.0157522 (PMC4912118; doi:10.1371/journal.pone.0157522)
Supplement: S4 Table — (DOCX) [file pone.0157522.s014.docx]

Table S4. List of genes identified from meta-analysis which are shared across multiple individual stresses.

| **Sl. No** | **Helianthus Gene ID** | **Gene name** | **Full name of the gene** | **Unigene ID** |
| --- | --- | --- | --- | --- |
|  | | **Upregulated genes** | | |
| 1 | Heli034867_st | C2H-ZF | Cys2-His2 zinc finger protein | Q1H8M0 |
| 2 | Heli034296_st | Chitinase | Chitinase-like protein | A0A3A1 |
| 3 | Heli014011_st | MYB TF | Myb-related protein (MYB transcription factor) | O23160 |
| 4 | Heli039279_x_st | LTP | Non-specific lipid-transfer protein precursor (LTP) | NLTP |
| 5 | Heli053939_st | MI-1-PS | Myo-inositol 1-phosphate synthase | Q5W1H9 |
| 6 | Heli034630_st | MYC2 | Myc2 bHLH protein | A6NAB4 |
| 7 | Heli022643_st | CdCIPK2 | Calcium-dependent calmodulin-independent protein kinase isoform 2 | Q7XZK4 |
| 8 | Heli014421_x_st | LRR | F-box/FBD/LRR-repeat protein | FDL1 |
| 9 | Heli021474_st | Helicase | Helicase, C-terminal | A4PPX5 |
| 10 | Heli039539_st | PR5-1 | Pathogenesis-related 5-1 | Q8LSM9 |
| 11 | Heli012193_st | ERF12 | Ethylene-responsive transcription factor 12 (EREBP-12) | ERF81 |
| 12 | Heli040932_st | DEAD-Helicase | DEAD/DEAH box helicase, N-terminal | Q1RTU9 |
| 13 | Heli010651_st | PAP-1 | Purple acid phosphatase 1 | Q6J5M7 |
| 14 | Heli034227_st | LEA14 | LEA14_GOSHI Late embryogenesis abundant protein Lea14-A | LEA14 |
| 15 | Heli003083_st | ERD6 | Sugar transporter ERD6-like | EDL16 |
|  |  |  | **Downregulated genes** |  |
| 16 | Heli000419_x_st | DnaJ | DnaJ-like protein | Q38HT9 |
| 17 | Heli001720_st | PSI center | Photosystem I reaction centre subunit N | Q1SQB6 |
| 18 | Heli000803_st | Chaperonin21 | Chloroplast chaperonin 21 | Q6B4V4 |
| 19 | Heli028659_st | BA-P12 | Putative blight-associated protein p12 | Q6K4C6 |
| 20 | Heli005046_st | ARP8c | Autophagy-related protein 8C precursor (Autophagy-related ubiquitin-like modifier) | ATG8C |
| 21 | Heli003783_x_st | O-6FAD | Omega-6 fatty acid desaturase (Microsomal oleic acid desaturase) | Q9LLL7 |
| 22 | Heli013583_x_st | H2A | Histone H2A | Q677E7 |
| 23 | Heli004029_x_st | RL10 | Envelop glycoprotein RL10 | Q910I9 |
| 24 | Heli021470_st | Mg chelatase | Protoporphyrin IX:Mg chelatase | Q07893 |
| 25 | Heli055964_st | GyraseA | DNA gyrase subunit A, chloroplast/mitochondrial precursor | GYRA |
| 26 | Heli055946_st | DNA TOPII | DNA topoisomerase II (EC 5.99.1.3) | Q8GSC4 |
| 27 | Heli001044_x_st | LHCP | Light-harvesting chlorophyll a/b-binding protein (LHCP) | Q40247 |
| 28 | Heli048682_st | HxK2 | Hexokinase-2, chloroplast precursor (EC 2.7.1.1) (NtHxK2) | HXK2 |
| 29 | Heli008947_x_st | TiPa | Aquaglyceroporin (Tonoplast intrinsic protein (Tipa)) | Q9XG70 |
| 30 | Heli005472_x_st | Cys-γ syn | Cystathionine gamma synthase | Q6R8F6 |
