# Supplementary material for: Transcriptome Analysis of Sunflower Genotypes with Contrasting Oxidative Stress Tolerance Reveals Individual- and Combined- Biotic and Abiotic Stress Tolerance Mechanisms
Source: PLoS One. 2016 Jun 17;11(6):e0157522. doi: 10.1371/journal.pone.0157522 (PMC4912118; doi:10.1371/journal.pone.0157522)
Supplement: S5 Table — (DOCX) [file pone.0157522.s015.docx]

Table S5. Effect of different concentrations of menadione on sunflower seedling survival and recovery growth.

| **Concentration of menadione (mM)** | | **Durations of incubation in menadione (min)** | | | | | | |
| --- | --- | --- | --- | --- | --- | --- | --- | --- |
|  | | **30** | | **60** | | **120** | | **180** |
| Seedling survival (%)* | | | | | | | | |
| **1** | | 100 | | 100 | | 100 | | 100 |
| **2** | | 100 | | 70 | | 65 | | 50 |
| **3** | | 50 | | 50 | | 16.6 | | 16.6 |
| **4** | | 33.33 | | 40 | | 0 | | 0 |
| **5** | | 20 | | 16.66 | | 0 | | 0 |
| Reduction in growth (%)** | | | | | | | | |
| **0.25** | 36.45 | | 71.43 | | 92.95 | | 94.34 | |
| **0.5** | 70.14 | | 77.14 | | 87.77 | | 91.54 | |
| **1** | 80.28 | | 81.69 | | 96.17 | | 93.4 | |
| **2** | 89.63 | | 90 | | 98 | | 100 | |
| **CD** | 8.53 | | 5.65 | | 5 | | 1.35 | |

*sunflower (var. Morden) seedlings were directly exposed to different concentrations of menadione for varying durations. After the treatment, seedlings were allowed to recover at 30 ^o^C with 60% relative humidity for 72 h in water. As a measure of oxidative stress damage, per cent survival (n=12) was recorded at the end of recovery period. The values are mean of three independent experiments.

** Recovery growth was measured as per cent reduction in seedling growth over control (n=12) at the end of recovery period. The values are mean of three independent experiments.
