# Supplementary material for: Transcriptome Analysis of Sunflower Genotypes with Contrasting Oxidative Stress Tolerance Reveals Individual- and Combined- Biotic and Abiotic Stress Tolerance Mechanisms
Source: PLoS One. 2016 Jun 17;11(6):e0157522. doi: 10.1371/journal.pone.0157522 (PMC4912118; doi:10.1371/journal.pone.0157522)
Supplement: S6 Table — (DOCX) [file pone.0157522.s016.docx]

Table S6. Response of sunflower genotypes to menadione induced oxidative stress.

| Genotype response | No. of genotypes |
| --- | --- |
| Resistant or Strong | 27 |
| Moderate resistant | 14 |
| Sensitive | 8 |
| Extremely sensitive | 6 |

Genotypes were categorized based on survival (%) and reduction in recovery growth (%). Resistant: if the survival is 75-100 %, Moderate resistant: 50-75 % survival and recovery, sensitive: 10-50% survival and recovery, extremely sensitive: Seedlings did not survive after menadione treatment.
